# Cluster Target Tracking Based on Multi-Sensor Adaptive GLMB Filter

**DOI:** 10.3390/s26113559

**Published:** 2026-06-03

**Authors:** Zheng Zhang, Daozhi Wei, Xirui Xue

**Affiliations:** 1 Graduate College, Air Force Engineering University, Xi’an 710051, China; kiukiuyouxiang@outlook.com; 2 Air and Missile Defense College, Air Force Engineering University, Xi’an 710051, China; superwsw2012@163.com

**Keywords:** cluster target tracking, MS-AGLMB filter, Gibbs sampling, CPHD filter, virtual leader-follower model

## Abstract

**Highlights:**

**What are the main findings?**

- We embed an adaptive CPHD filter into the GLMB filtering framework, which enables estimation of detection probability and clutter rate from the GLMB posterior.
- We introduce the virtual leader–follower cluster structure model into the MS-GLMB filtering framework, such that the states of other cluster members are taken into account when estimating the state of each cluster member.

**What are the implications of the main findings?**

- Through real-time online estimation of detection probability and clutter rate, the proposed algorithm enables robust target tracking in scenarios with unknown environmental parameters.
- The introduction of the virtual leader–follower cluster model equips our algorithm with the capability to estimate cluster states, leading to more accurate estimation of cluster targets.

**Abstract:**

In complex detection environments, unknown detection probability and clutter rate hinder accurate tracking of cluster targets. To address this issue, this paper proposes a novel multi-sensor adaptive generalized labeled multi-Bernoulli (MS-AGLMB) filter. Specifically, we consider interactions among cluster members and adopt a virtual leader–follower model to describe cluster kinematics. Given unknown environmental parameters, we employ an adaptive cardinalized probability hypothesis density (CPHD) filter to estimate the detection probability and clutter rate in real time. Furthermore, we use Gibbs sampling to efficiently truncate GLMB association hypotheses, obtaining the posterior density and solving the multi-sensor measurement partitioning problem. A joint prediction and update strategy enables simultaneous estimation of target trajectories, detection probability, clutter rate, and cluster structure. Simulation results demonstrate that the proposed algorithm achieves greater robustness in scenarios with time-varying detection probability and clutter rate, outperforming comparison filters in cluster target tracking.

## 1. Introduction

With the rapid development of sensor technology, cluster target tracking is expanding from military applications to a wide range of civilian domains, including crowd monitoring and management [1], unmanned aerial vehicle control [2], and biomedical research [3]. Its objective is to extract target state information to support prediction, decision-making, and control. However, in complex detection environments, sensor measurements are subject to high-intensity, non-uniform noise and clutter, which severely degrade the performance of traditional fixed-threshold detection methods [4]. This issue is particularly pronounced for dense cluster targets, often resulting in missed or false tracks and misidentification of cluster structure, ultimately rendering subsequent tracking algorithms ineffective [5].

When tracking a cooperative drone formation, members typically exhibit similar motion patterns. A decline in communication quality or the presence of adversarial interference can cause a sudden drop in radar detection probability and a sharp increase in clutter [6]. In public space surveillance systems, pedestrians naturally form cohesive groups. The number of individuals is typically unknown a priori, and the detection probability of visual or radar sensors varies with changes in crowd density, occlusion, and lighting [7]. In multi-radar air defense networks, targets such as aircraft formations or missile salvos appear as distinguishable clusters. Due to electronic countermeasures, sensor performance can rapidly degrade, resulting in unknown and time-varying clutter rates and detection probabilities [8].

Owing to sensor resolution limitations, cluster target tracking is typically categorized into three types: resolvable, unresolvable, and partially resolvable. When the sensor is distant from the cluster or has poor resolution, measurements may overlap significantly, preventing the discrimination of individual cluster members. In such cases, the cluster can be modeled as an extended target based on the consistent motion of its members, using parametric models such as ellipses, star-convex shapes, or random hypersurface models (RHMs). Research in this area focuses on cluster shape evolution and dynamic behaviors such as splitting and merging [9]. Conversely, when the sensor is close to the cluster or has high resolution, tracking the states of individual members becomes feasible. Unlike general multi-target tracking, this scenario requires explicit consideration of cluster structure and cooperative interactions among members. Structural models such as the virtual leader model, Markov random field (MRF) model, and evolutionary network model have been proposed and widely applied in this situation [10].

Filtering methods based on random finite set (RFS) theory have been widely developed and applied because they avoid explicit data association while accurately modeling multi-target states, aligning well with practical tracking scenarios. Representative RFS-based filters include the probability hypothesis density (PHD) filter, the multi-target multi-Bernoulli (MeMBer) filter, and the Poisson multi-Bernoulli mixture (PMBM) filter [11]. To address the inability of conventional RFS-based filters to maintain target identity and output distinguishable trajectories, labeled RFS-based filters have been developed. In labeled RFS formulations, each target state is augmented with a unique label, which enables the filter to establish and preserve the identity of individual objects across time [12]. This identity information is essential for forming continuous trajectories, especially in cluster tracking scenarios where maintaining the association between targets and their trajectories is critical for analyzing group structure and interaction. Ba-Ngu Vo et al. [13] used a tractable update approximation to avoid cardinality errors; the resulting labeled multi-Bernoulli (LMB) filter outputs estimated trajectories and achieves better performance than the MeMBer filter. The generalized labeled multi-Bernoulli (GLMB) filter, a generalization and extension of the LMB filter [14,15], offers greater flexibility in target birth, death, and state transitions, making it more suitable for complex cluster target tracking. Based on this, Punchihewa et al. [16] incorporated a jump Markov system (JMS) to model different motion modes, enabling the GLMB filter to track maneuvering targets. To reduce computational complexity, ref. [17] introduced a joint prediction and update framework with a Gibbs sampling truncation strategy, which reduces the number of truncations and decomposes the high-dimensional measurement association problem into lower-dimensional conditional sampling. Furthermore, with the adoption of tempered Gibbs sampling [18], the computational efficiency of the GLMB filter is further improved. Its computational complexity is reduced from cubic order to linear order. This approach not only enhances the convergence of the sampling process but also addresses the real-time performance bottleneck of GLMB filtering.

Using multiple sensors for target tracking can theoretically improve the certainty of state estimation, thereby enhancing tracking performance [19,20]. Multi-sensor target tracking typically employs centralized or distributed architectures. In centralized multi-sensor GLMB tracking, [21] proposed a sensor-order-independent, low-complexity fusion scheme that reduces computational complexity to the product of the number of measurements per sensor, making it well suited for heterogeneous data fusion. For distributed multi-sensor GLMB tracking, research has focused on fusion rules, label consistency, and computational efficiency. Fantacci et al. [22] proposed arithmetic average fusion, preserving the GLMB closed form and robustness. Li et al. [23] addressed label inconsistency among distributed nodes via label matching and generalized covariance intersection (GCI) fusion. Li et al. [24] further introduced average consensus and LMB approximation to improve computational efficiency in large-scale networks.

However, the use of GLMB filters for tracking resolvable cluster targets has not yet been explored. Existing RFS-based cluster tracking methods generally assume that the statistical characteristics of the measurement environment are known a priori, focusing on measurement association and state estimation of detected targets, with limited investigation into maintaining continuous tracking under dynamically changing measurement noise. Traditional fixed-threshold detection methods are inadequate for handling variations in noise and clutter statistics and lack adaptability to non-uniform noise environments. Although some single-sensor studies have addressed these challenges [25,26,27,28], the multi-sensor cluster tracking domain still lacks a joint detection and tracking method that adapts to environmental noise and clutter while accounting for the specific characteristics of cluster targets [29].

To improve multi-sensor tracking of cluster targets in unknown measurement environments, this paper proposes an adaptive multi-sensor GLMB filter. The main contributions of this paper are as follows.

1. Cluster structure-aware GLMB filtering: We embed a virtual leader–follower model into the GLMB prediction, so that the motion of each cluster member is conditioned on the estimated cluster center. This replaces the standard independent motion assumption that would otherwise cause kinematic inconsistency within a cluster.
2. Adaptive parameter estimation embedded in a labeled RFS filter: We construct an augmented state space and run an adaptive CPHD filter that inherits its prior from the current GLMB posterior. This closes the loop between trajectory-level filtering and parameter estimation in a multi-sensor setup—unlike existing single-sensor adaptive CPHD filters, our design estimates clutter rate and detection probability from the same labeled posterior that is used for trajectory output.
3. Efficient multi-sensor hypothesis truncation via Gibbs sampling: We formulate the joint prediction–update with a multi-sensor measurement partition that is solved by Gibbs sampling, exploiting a suboptimal Markov approximation to reduce the sampling complexity from product to sum. This makes the joint multi-sensor assignment tractable for an adaptive GLMB filter that must also carry augmented parameter states.

The three components above are integrated so that the CPHD-estimated parameters feed the multi-sensor likelihood model, the cluster structure guides the prediction, and Gibbs sampling keeps the hypothesis set manageable. This produces a filter that simultaneously outputs labeled trajectories, cluster structure, and environmental parameters—a combination not present in prior adaptive RFS filters, multi-sensor GLMB filters, or existing cluster/group tracking methods.

The remainder of this paper is structured as follows. Section 2 introduces RFS theory and the GLMB filter prediction and update steps. Section 3 presents the system model for cluster target tracking using the GLMB filter. Section 4 describes the proposed multi-sensor adaptive GLMB filter, focusing on the adaptive CPHD-based estimation of detection probability and clutter rate, and the multi-sensor measurement partition. In Section 5, we designed three experimental scenarios to evaluate the tracking accuracy of the proposed filter and to discuss its tracking performance under different measurement conditions. Section 6 concludes this paper.

## 2. Background

### 2.1. Theory of Random Finite Sets

Within the framework of the RFS, the number of elements in the set is a random variable with a finite range of values, and each element itself is also a random variable, with no inherent ordering among the elements. Therefore, in multi-target tracking, the states of multiple targets and sensor measurements can naturally be unified and characterized using an RFS [30].

The single-target state and its label are denoted as x and x, respectively, while the multi-target state and its label are denoted as X and X, respectively. The single-target state space, label space, and measurement space are denoted as X, L, and Z, respectively. We use F(S) to denote all finite subsets of the set S. The cardinality of a set is defined as ${\left[h(\cdot )\right]}^{X}=\prod_{x\in X}h(x)$, and the inner product of the two functions f and g is defined as 〈f, g〉≜∫f(x)g(x)dx. The arbitrary parameter form of the Kronecker delta function is defined as

(1) $$\delta_{S}(X)=\left\{\begin{array}{ll} 1, & X=S\\ 0, & X\neq S \end{array}\right.,$$

and the indicator function is defined as

(2) $$1_{S}(X)=\left\{\begin{array}{ll} 1, & \;X\subseteq S\\ 0, & \;\mathrm{otherwise}\; \end{array}\right..$$

The state of a label’s single target can be represented as x=(x, l)∈X×L. Typically, each label l at time k is an ordered pair $l=\left(t_{b},\;i\right)$, where $t_{b}\leq k$ represents the birth time, and i is a unique index used to distinguish targets born simultaneously. Let $B_{k-1}$ denote the birth label space at the current time k−1. Then, the birth label at time k belongs to the label space $B_{k}=\{(k,\;i):\;i\in N\}$, so $L_{k-1}\cap B_{k}=\emptyset$. The label space at time k becomes $L_{k}=L_{k-1}\cup B_{k}$.

The label indicator is defined as

(3) $$\Delta (X)=\delta_{\left|X\right|}(|L(X)|)$$

where |X| denotes the cardinality of the label set X, and L:X×L→L is a mapping from the label RFS to the labels, satisfying the projection L(x, l)=l. Different label indicators are used to ensure that X has distinct labels.

The integral F(X×L)→R of the function f is defined as

(4) $$\int f(X)\delta X=\sum_{i=0}^{\infty }\frac{1}{i!}\sum_{\left(l_{1},\ldots ,l_{i}\right)\in L^{i}}\int_{X^{i}}f\left(\left\{\left(x_{1},\;l_{1}\right),\ldots ,\left(x_{i},\;l_{i}\right)\right\}\right)d\left(x_{1},\ldots ,\;x_{i}\right).$$

In multi-target Bayesian filtering with labels, with the multi-target transition density $f_{k}$ and the measurement likelihood function $g_{k}$, the probability density function $\pi_{k}$ of the multi-target labeled state propagates over time via Bayesian recursion [31], expressed as follows

(5) $$\pi_{k|k-1}(X_{k})=\int f_{k}\left(X_{k}|X_{k-1}\right)\pi_{k-1}(X_{k-1})\delta X_{k-1}$$

(6) $$\pi_{k|k}\left(X_{k}\right)=\frac{g_{k}\left(Z_{k}|X_{k}\right)\pi_{k|k-1}(X_{k})}{\int g_{k}\left(Z_{k}|X_{k}\right)\pi_{k|k-1}(X_{k})\delta X_{k}}$$

where (5) and (6) use set integrals, as defined in (4).

### 2.2. GLMB Filter

Let the multi-target state at time k−1 be described by a GLMB RFS $X_{k-1|k-1}$, whose posterior probability density is $\pi_{k-1|k-1}$. The target is observed by V independent sensors, which generate a set of measurements at time k. The objective of this filter is to estimate the density $\pi_{k|k}$ of the RFS $X_{k|k}$ given all measurements up to time k. Measurement association is performed via a multidimensional assignment algorithm during the update step. Thus, the multi-target state can be obtained through the prediction and update steps of Bayesian recursion.

#### 2.2.1. Prediction

The posterior GLMB density at time k−1 is given by

(7) $$\pi_{k-1|k-1}(X_{k-1})=\Delta (X_{k-1})\sum_{(I,\xi )\in F(L)\times \Xi }\omega_{k-1|k-1}^{\left(I,\xi \right)}\delta_{I}(L(X_{k-1})){\left[p_{k-1|k-1}^{\left(\xi \right)}\right]}^{X_{k-1}}$$

where I⊆L is the label subset, ξ∈Ξ is the historical association mapping index, $\omega_{k-1|k-1}^{\left(I,\xi \right)}\geq 0$ is the assumed weight satisfying $\sum_{I,\xi }\omega_{k-1|k-1}^{\left(I,\xi \right)}=1$, and $p_{k-1|k-1}^{\left(\xi \right)}(x,\;l)$ is the single-target state probability density function corresponding to the label l.

The prediction step involves predicting surviving targets and introducing new targets. Let the target survival probability be $p_{S}(x_{k-1},\;l_{k-1})$, and the state transition density be $f_{k|k-1}\left(x_{k}|x_{k-1},\;l_{k-1}\right)$. For surviving targets, given the assumption (I, ξ), the prediction density for each label l∈I is

(8) $$p_{S,k|k-1}^{\left(\xi \right)}\left(x_{k},\;l_{k}\right)=\frac{\langle p_{S}(x_{k-1},\;l_{k-1})f_{k|k-1}\left(x_{k}|x_{k-1},\;l_{k-1}\right),p_{k-1|k-1}^{\left(\xi \right)}(x_{k-1},\;l_{k-1})\rangle }{\eta_{S}^{\left(\xi \right)}(l)}$$

where $\eta_{S}^{\left(\xi \right)}(l)=\langle p_{S}(x_{k-1},\;l_{k-1}),p_{k-1|k-1}^{\left(\xi \right)}(x_{k-1},\;l_{k-1})\rangle$ is the survival probability for label l, and the corresponding survival assumption weight is

(9) $$\omega_{S,k|k-1}^{\left(I,\xi \right)}=\omega_{k-1|k-1}^{\left(I,\xi \right)}\prod_{l\in I}\eta_{S}^{\left(\xi \right)}(l).$$

Newborn targets are described using an LMB process with a density of

(10) $$\pi_{\gamma ,k}(X_{k})=\Delta (X_{k})\sum_{J\subseteq B_{k}}\omega_{\gamma ,k}^{\left(J\right)}\delta_{J}(L(X_{k})){\left[p_{\gamma ,k}(x_{k},\;l_{k})\right]}^{X_{k}}$$

where $\omega_{\gamma ,k}^{\left(J\right)}$ is the weight of the newborn label set J, and $p_{\gamma ,k}(x_{k},\;l_{k})$ is the state density of the newborn target.

Since the survival target is independent of the newborn target, the predicted GLMB density is the convolution of the two, i.e., a combination of all possible survival label sets I, newborn label sets J, and historical indices ξ:

(11) $$\pi_{k|k-1}(X_{k})=\Delta (X_{k})\sum_{I,J,\xi }\omega_{k|k-1}^{\left(I,J,\xi \right)}\delta_{I\cup J}(L(X_{k})){\left[p_{k|k-1}^{\left(\xi \right)}\right]}^{X_{k}}$$

where the weights are $\omega_{k|k-1}^{\left(I,J,\xi \right)}=\omega_{S,k|k-1}^{\left(I,\xi \right)}\cdot \omega_{\gamma ,k}^{\left(J\right)}$. For label l∈I, the density is $p_{S,k|k-1}^{\left(\xi \right)}(x_{k},\;l_{k})$, for label l∈J, the density is $p_{\gamma ,k}(x_{k},\;l_{k})$.

To simplify the expression, the predicted density is rewritten in standard GLMB form

(12) $$\pi_{k|k-1}(X_{k})=\Delta (X_{k})\sum_{(\tilde{I},\tilde{\xi })\in F(L)\times \dot{\Xi }}\omega_{k|k-1}^{\left(\tilde{I},\tilde{\xi }\right)}\delta_{\tilde{I}}(L(X_{k})){\left[p_{k|k-1}^{\left(\tilde{\xi }\right)}\right]}^{X_{k}}$$

where $\tilde{I}=I\cup J$, $\tilde{\xi }$ encodes the information of (I,J,ξ), and $\tilde{\Xi }$ is the corresponding index set.

#### 2.2.2. Update

Given the prior density $\pi_{k|k-1}$ and the multi-sensor measurement set $Z_{k}$, the posterior GLMB density is obtained via Bayesian updating. Since the prior density is in GLMB form and the multi-sensor likelihood is factorizable, the posterior density remains a GLMB,

(13) $$\pi_{k|k}\left(X_{k}|Z_{k}\right)\propto \Delta (X_{k})\sum_{\left(\tilde{I},\tilde{\xi }\right)}\sum_{\theta \in \Theta (\tilde{I})}\omega_{k|k}^{\left(\tilde{I},\tilde{\xi },\theta \right)}\delta_{\tilde{I}}(L(X_{k})){\left[p_{k|k}^{\left(\tilde{\xi },\theta \right)}(x_{k},\;l_{k})\right]}^{X_{k}},$$

where $\omega_{k|k}^{\left(\tilde{I},\tilde{\xi },\theta \right)}$ are the update weights, and $p_{k|k}^{\left(\tilde{\xi },\theta \right)}(x_{k},\;l_{k})$ is the updated single-target density.

For each prediction hypothesis $\left(\tilde{I},\;\tilde{\xi }\right)$ and association mapping θ, define the multi-sensor likelihood function. For label l, given $\theta (l)=\left(j_{1},\ldots ,\;j_{V}\right)$, the likelihood is

(14) $$\psi_{k}^{\left(\theta (l)\right)}(x_{k},\;l_{k})=\prod_{v=1}^{V}\left[\delta_{0}\left(j_{v}\right)q_{D}^{\left(s\right)}(x_{k},\;l_{k})+\left(1-\delta_{0}\left(j_{v}\right)\right)\frac{p_{D}^{\left(v\right)}(x_{k},\;l_{k})g^{\left(v\right)}\left(z_{k,j_{v}}^{\left(v\right)}|x_{k},\;l_{k}\right)}{\kappa^{\left(v\right)}\left(z_{k,j_{v}}^{\left(v\right)}\right)}\right],$$

where $p_{D}^{\left(v\right)}(x_{k},\;l_{k})$ is the detection probability of sensor v, $q_{D}^{\left(v\right)}(x_{k},\;l_{k})=1-p_{D}^{\left(v\right)}(x_{k},\;l_{k})$ is the false negative probability, $g^{\left(v\right)}(z|x_{k},\;l_{k})$ is the measurement likelihood of sensor v, $\kappa^{\left(v\right)}(z)$ is the noise intensity function of sensor v, typically modeled as a Poisson process, i.e., $\kappa^{\left(v\right)}(z)=\lambda_{c}^{\left(v\right)}c^{\left(v\right)}(z)$, $\lambda_{c}^{\left(v\right)}$ is the noise rate, and $c^{\left(v\right)}(z)$ is the spatial distribution of noise.

If the target is not detected by any of the sensors (i.e., all $j_{v}=0$), then

(15) $$\psi_{k}^{\left(\theta (l)\right)}(x_{k},\;l_{k})=\prod_{v=1}^{V}q_{D}^{\left(v\right)}(x_{k},\;l_{k}),$$

where the updated single-target density equals the predicted density times the multi-sensor likelihood, normalized as

(16) $$p_{k|k}^{\left(\tilde{\xi },\theta \right)}(x_{k},\;l_{k})=\frac{p_{k|k-1}^{\left(\tilde{\xi }\right)}(x_{k},\;l_{k})\psi_{k}^{\left(\theta (l)\right)}(x_{k},\;l_{k})}{\eta^{\left(\tilde{\xi },\theta \right)}(l)},$$

where the normalization constant is $\eta^{\left(\tilde{\xi },\theta \right)}(l)=\int p_{k|k-1}^{\left(\tilde{\xi }\right)}(x_{k},\;l_{k})\psi_{k}^{\left(\theta (l)\right)}(x_{k},\;l_{k})dx$.

The updated weights are proportional to the product of the predicted weights and the likelihood factor

(17) $$\omega_{k|k}^{\left(\tilde{I},\tilde{\xi },\theta \right)}\propto \omega_{k|k-1}^{\left(\tilde{I},\tilde{\xi }\right)}\prod_{l\in \tilde{I}}\eta^{\left(\tilde{\xi },\theta \right)}(l_{k}).$$

Finally, the above recursive steps can be combined into a single prediction-updating posterior GLMB density

(18) $$\pi_{k}\left(X_{k}|Z_{k}\right)\propto \Delta \left(X_{k}\right)\sum_{I,\xi ,I,\theta }\omega^{\left(I,\xi \right)}\omega_{Z}^{\left(I,\xi ,I,\theta \right)}\delta_{I}\left[L\left(X_{k}\right)\right]{\left[p_{Z}^{\left(\xi ,\theta \right)}\right]}^{X_{k}}.$$

Due to the rapid growth in the number of hypotheses, pruning and merging must be performed to retain the hypotheses with the highest weights and merge similar spatial distributions, thereby controlling computational complexity. Finally, target state estimates are extracted from the posterior GLMB density by selecting labels with a probability of existence above a threshold, taking the mean of their state distribution as the estimated target state, and using the label history to form a trajectory. Under the standard assumptions of the GLMB filter, the state of the newly detected target $X_{B}$ is known, as are the detection probability $p_{D}$ and the noise rate $\lambda_{c}$. Next, in Section 4.2, we will introduce the adaptive CPHD filter, and in Section 4.3 and Section 4.4, we will treat $p_{D}$ and $\lambda_{c}$ as independent parameters to be incorporated into the GLMB iteration process using the CPHD filter.

## 3. Tracking System Model

### 3.1. Modeling of Cluster Structures

**Figure 1:**
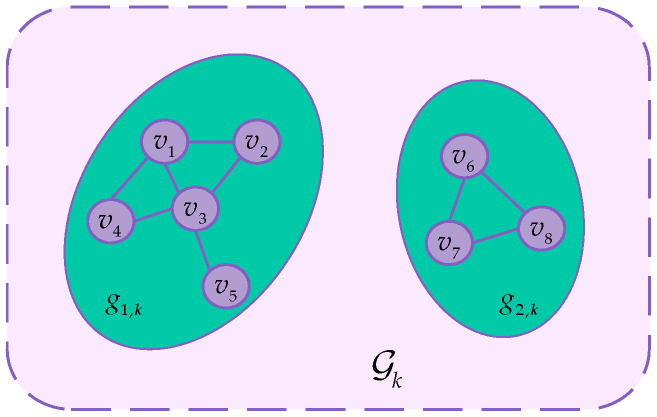
Cluster undirected graph structure.

In RFS-based cluster target tracking theory, effectively describing the cluster structure enables unified modeling of both the cluster’s overall state and the individual states of its members, thereby helping the filter capture the dynamic evolutionary characteristics of the cluster. This paper employs graph theory to provide a mathematical representation of the dynamic interactions within the cluster, thereby laying the theoretical foundation for cluster Bayesian filtering [32]. The topological structure of the cluster at any given time k can be defined as a labeled undirected graph $G_{k}=\left(V_{k},\;E_{k}\right)$, as shown in Figure 1. The set $V_{k}=\left\{v_{1},\;v_{2},\ldots ,\;v_{N_{k}}\right\}$ represents all $N_{k}$ members of the cluster at time k. Each vertex $v_{i}$ is associated with a unique label $l_{i}$, which is typically an ordered pair $\left(t_{b},\;i\right)$, where $t_{b}$ denotes the target’s birth time and i serves as a unique identifier to ensure the continuity of the trajectory throughout the tracking process. Each vertex represents the corresponding target state estimate $x_{i}^{k}$ and its error covariance matrix $P_{i}^{k}$. The set $E_{k}\subseteq V_{k}\times V_{k}$ defines the connection relationships between vertices. An edge $e_{ij}=\left(v_{i},\;v_{j}\right)\in E_{k}$ indicates that targets i and j belong to the same cluster at time k. The graph is undirected, i.e., $e_{ij}=e_{ji}$, reflecting the symmetry of interactions among members.

The existence of an edge is determined by a state-based decision function. This paper adopts the Mahalanobis distance as a measure of statistical similarity between two target states, which accounts for the uncertainty in the estimation of each target state. The Mahalanobis distance between vertices $v_{i}$ and $v_{j}$ is defined as

(19) $$d_{ij}^{k}=\sqrt{{\left(x_{i}^{k}-x_{j}^{k}\right)}^{T}{\left(P_{i}^{k}+P_{j}^{k}\right)}^{-1}\left(x_{i}^{k}-x_{j}^{k}\right)}.$$

Based on this, the adjacency matrix $A_{k}$ of the graph is constructed as follows

(20) $$A_{k}[i,\;j]=\eta (i,\;j)=\left\{\begin{array}{ll} 1, & \;\mathrm{if}\;d_{ij}^{k}\leq T_{\eta }\;\mathrm{and}\;i\neq j\\ 0, & \;\mathrm{otherwise}\; \end{array}\right.$$

where $T_{\eta }$ is a predefined distance threshold. When the statistical distance between two target states is sufficiently close, they are deemed to interact and are thus connected by an edge in the graph structure. Once the adjacency matrix $A_{k}$ is determined, the problem of identifying discrete clusters from the graph $G_{k}$ is transformed into the problem of extracting connected components in graph theory. A connected component is a maximal connected subgraph of a graph in which there is a path between any two vertices; each identified connected component corresponds to an independent cluster.

### 3.2. Cluster Target Movement Model

The virtual leader–follower model [33] uses Markov random fields (MRFs) and repulsive forces to simulate cluster motion, possessing the ability to accurately represent the cluster structure over time. In the virtual leader–follower model, the motion of each member in the cluster is influenced by a virtual leader. In the cluster set $C=\left\{C_{1},\;C_{2},\ldots ,\;C_{M}\right\}$, for a target belonging to the cluster $C_{m}$, its equation of motion is

(21) $$x_{l,k}=F_{k}x_{l,k-1}+B_{k}x_{C_{m}}^{v}+w_{l,k-1}$$

where $w_{l,k-1}\sim N\left(0,\;Q_{k-1}\right)$ is the process noise, $x_{C_{m}}^{v}=\sum_{l\in L_{C_{m}}}x_{l,k-1}/\left|C_{m}\right|$ is the virtual leader, the superscript v denotes virtual, $C_{m}\subset L$ and $\cup_{m=1}^{M}C_{m}\subseteq L$, the target label l belongs to the cluster $C(l)=C_{m}$, the virtual leader state of cluster $C_{m}$ is $x^{v}=\left(x^{v},\;l^{v}\right)\in X\times L^{v}$, the virtual leader label is $l^{v}=\left(k_{b}^{v},\;i^{v},\;C_{m}\right)\in L^{v}\subseteq L$, $k_{b}$ is the birth time, and i is the unique index.

The transition matrix $F_{k}$ and the cluster coupling matrix $B_{k}$ are

(22) $$F_{k}=\left[\begin{array}{cc} I_{2} & \Delta tI_{2}\\ O_{2} & I_{2} \end{array}\right],\;B_{k}=\left[\begin{array}{cc} O_{2} & \frac{\Delta t}{\left|C_{m}\right|}I_{2}\\ O_{2} & O_{2} \end{array}\right]$$

where $I_{2}$ is a 2-dimensional identity matrix, and $O_{2}$ is a 2-dimensional zero matrix. For independent targets that do not belong to any cluster, we have

(23) $$x_{l,k}=F_{k-1}x_{l,k-1}+w_{l,k-1}.$$

With the cluster center of mass serving as the virtual leader, its dynamics are

(24) $$x_{l^{v},k}^{v}={\bar{F}}_{k-1}x_{l^{v},k-1}^{v}+{\bar{w}}_{l^{v},k-1},$$

In particular, ${\bar{F}}_{k-1}$ is usually the same as $F_{k-1}$, and ${\bar{w}}_{l^{v},k-1}\sim N\left(0,\;{\bar{Q}}_{k-1}\right)$.

Given the current multi-target state $X_{k-1}$, the transition density for the next time step is

(25) $$f_{k|k-1}\left(X_{k}|X_{k-1}\right)=\sum_{X_{S,k}⊎X_{B,k}=X_{k}}f_{S}\left(X_{S,k}|X_{k-1}\right)f_{B}\left(X_{B,k}\right),$$

where $X_{S,k}$ is the set of surviving targets, $X_{B,k}$ is the set of newly generated targets, and ⊎ denotes the disjoint union.

The transition density for surviving targets is

(26) $$f_{S}\left(X_{S,k}|X_{k-1}\right)=\Delta \left(X_{S,k}\right)\Delta \left(X_{k-1}\right)1_{L\left(X_{k-1}\right)}\left(L\left(X_{S,k}\right)\right)\times \prod_{x_{k-1}\in X_{k-1}}\phi_{S}\left(X_{S,k}|x_{k-1}\right),$$

where the single-target transition likelihood function is expressed as follows

(27) $$\phi_{S}\left(X_{S,k}|x_{k-1}\right)=\left\{\begin{array}{ll} q_{S}\left(x_{k-1},\;l_{k-1}\right), & \mathrm{if}\;l_{k-1}\notin L\left(X_{S,k}\right)\\ p_{S}\left(x_{k-1},\;l_{k-1}\right)f_{k|k-1}\left(x_{k}|x_{k-1},\;l_{k-1}\right), & \mathrm{if}\;\left(x_{k},\;l_{k-1}\right)\in X_{S,k} \end{array}\right.,$$

where label mapping $L(X_{k-1})=\{L(x_{k-1}):x_{k-1}\in X_{k-1}\}$, label extraction operator $L(x_{k-1})=l_{k-1}$, $q_{S}(x_{k-1},\;l_{k-1})$ denotes the target extinction probability, $p_{S}(x_{k-1},\;l_{k-1})$ denotes the target survival probability, and the conditional transition density is $f_{k|k-1}\left(x_{k}|x_{k-1},\;l_{k-1}\right)$. For C≠∅, the cluster motion equation is given by (21). For C=∅, the cluster motion equation is given by (23).

### 3.3. Single-Sensor Cluster Target Observation Model

For target l in state x, its measurement generation model is

(28) $$z_{k-1}=h_{k-1}(x_{k-1})+v_{k-1},$$

where $h_{k-1}(x_{k-1})$ is the measurement function, $v_{k-1}\sim N\left(0,\;R\right)$ is the measurement noise, and R is the measurement noise covariance.

Given a multi-target state $X_{k-1}$, the likelihood function for the measurement set $Z_{k-1}$ is

(29) $$g_{k-1}\left(Z_{k-1}|X_{k-1}\right)=\sum_{\theta \in \Theta_{k}}\left[\prod_{(x,l)\in X_{k-1}}\psi_{Z}^{\left(\theta (l)\right)}(x,\;l)\right]{\lambda_{c}}_{k-1}\left(Z_{k-1}\backslash \left\{z_{\theta (l)}\right\}\right),$$

where θ:L→{0,1,…,|Z|}. Let c be an association map, $\Theta_{k-1}$ be the set of all possible association maps, and θ(l) be the measurement indices associated with the target l. If θ(l)=0, the target l is not detected (missed). If θ(l)=j>0, the target l is associated with the measurement $z_{j}$. Symbol $Z_{k-1}\backslash \left\{z_{\theta (l)}\right\}$ is the set of measurements remaining after removing the associated measurements, and ${\lambda_{c}}_{k-1}\left(\cdot \right)$ is the clutter intensity function, which follows the following Poisson process

(30) $${\lambda_{c}}_{k-1}(W)=e^{-\lambda_{c}}\prod_{z\in W}\lambda_{c}u(z),$$

where $\lambda_{c}$ is the average number of clutter points, and u(z) is a uniform distribution over the measurement space.

The likelihood of a single-target measurement associated in (29) is

(31) $$\psi_{Z_{k-1}}^{\left(j\right)}(x,\;l)=\{\begin{array}{ll} q_{D}(x,\;l), & j=0\\ p_{D}(x,\;l)g_{k-1}\left(z_{j}|x,\;l\right), & j>0 \end{array},$$

where $g_{k-1}\left(z_{j}|x,\;l\right)$ is the probability density of generating a measurement $z_{j}$ given the target state.

The virtual leader–follower model is most effective when cluster members maintain relatively stable and coordinated movement patterns. In such cases, adjusting each member’s dynamics to a shared cluster center effectively captures the kinematic coupling among members and prevents the filter from treating them as completely independent targets, thereby improving trajectory continuity and cluster structure estimates. Conversely, this model may become less suitable when the cluster structure is loose or highly dynamic. For loosely connected groups, members may exhibit weakly correlated motion, and the assumption of a single virtual leader per cluster may over-constrain the dynamics, leading to trajectory deviations. Additionally, during transients following fragmentation or coalescence, model mismatch may temporarily reduce estimation accuracy until the new clustering structure stabilizes.

## 4. MS-AGLMB Filter

**Figure 2:**
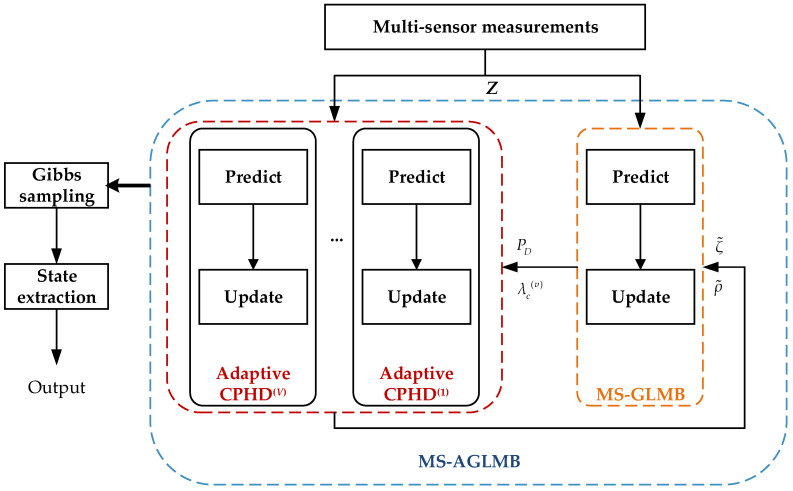
Flowchart.

In this section, we design a new cluster target tracking method based on the GLMB filter, capable of accurately tracking cluster targets in the absence of prior knowledge about the measurement environment. The filtering process is shown in Figure 2. First, an adaptive birth model is used to estimate the density of newly emerging targets, and a CPHD filter is employed to estimate the clutter rate and average detection probability, providing prior information for the multidimensional distribution of sensor measurements. Next, the estimated information is fed into a standard GLMB filter, which predicts target states based on cluster structure constraints. Gibbs sampling efficiently prunes high-weight association hypotheses, and the updated member states are used to update the cluster state and guide the prediction step for the next time step.

### 4.1. MS-GLMB Filter

The multi-sensor generalized labeled multi-Bernoulli (MS-GLMB) filter is an extension of the GLMB filter for multi-sensor scenarios. It is based on the theory of labeled RFS and achieves trajectory estimation by recursively propagating labeled multi-target densities. In this method, the MS-GLMB filter serves as the primary filtering module, and the required sensor clutter rate is provided by an embedded CPHD filter.

Consider a system with V sensors, assuming that detection events at each sensor are independent of one another. We model the detection probability as part of the target’s state, i.e., $x=\left(x,\;\alpha_{1},\;\alpha_{2},\ldots ,\;\alpha_{V},\;l\right)$, where the detection probability $\alpha_{v}\in [0,\;1]$ for each sensor $v^{th}$ varies over time. The state transition model described by (27) is rewritten as

(32) $$\phi_{S}\left(X_{S,k}|x_{k-1}\right)=\left\{\begin{array}{ll} q_{S}\left(x_{k-1},\;l_{k-1}\right), & \;\mathrm{if}\;l_{k-1}\notin L\left(X_{S,k}\right)\\ p_{S}\left(x_{k-1},\;l_{k-1}\right)\cdot f_{k|k-1}\left(x_{k}|x_{k-1},\;l_{k-1}\right)\cdot \prod_{v=1}^{V}f_{\alpha ,v}\left(\alpha_{k,v}|\alpha_{k-1,v}\right), & \;\mathrm{if}\;\left(x_{k},\;l_{k-1}\right)\in X_{S,k} \end{array}\right..$$

For multi-sensor likelihood, the measurement set $Z^{\left(v\right)}$ obtained by the v sensor may contain both measurements originating from the target and noise. The likelihood $g^{\left(v\right)}(z|x)$ that each target (x, a, l)∈X generates a measurement $z\in Z^{\left(v\right)}$ on sensor v is, and the detection probability is $a_{v}$. If the target is not detected, a measurement is not generated with probability $1-a_{v}$. The clutter term is modeled as a Poisson RFS, whose intensity ${\lambda_{c}}^{v}(z)$ is provided by the CPHD filter estimate. From (29), the multi-target likelihood function for a single sensor is

(33) $$g_{k-1}^{v}\left(Z_{k-1}^{\left(v\right)}|X_{k-1}\right)=\sum_{\theta^{\left(1\right)},\ldots ,\theta^{\left(v\right)}}\left[\prod_{(x,\alpha ,l)\in X_{k-1}}\left(\prod_{v=1}^{V}\psi_{Z_{k-1}^{\left(v\right)}}^{\left(\theta^{\left(v\right)}(l)\right)}\left(x,\;\alpha_{v},\;l\right)\right)\prod_{v=1}^{V}{\lambda_{c}}_{k-1}^{\left(v\right)}\left(Z_{k-1}^{\left(v\right)}\backslash \left\{z_{\theta^{\left(v\right)}(l)}\right\}\right)\right],$$

where $\theta^{\left(v\right)}:X\to Z^{\left(v\right)}\cup \{\emptyset \}$ is the association map on sensor $v^{th}$, and

(34) $$\psi_{Z_{k-1}^{\left(v\right)}}^{\left(j\right)}\left(x,\;\alpha_{v},\;l\right)=\left\{\begin{array}{ll} 1-\alpha_{v}, & j=0\\ \alpha_{v}\cdot g_{k-1}^{\left(v\right)}\left(z_{j}^{\left(v\right)}|x,\;l\right), & j>0 \end{array}\right..$$

Since the sensors are assumed to be independent of one another, the multi-sensor multi-target likelihood function can be written as the product of the individual sensor likelihoods

(35) $$g_{k-1}\left(Z_{k-1}|X_{k-1}\right)=\prod_{v=1}^{V}g_{k-1}^{\left(v\right)}\left(Z_{k-1}^{\left(v\right)}|X_{k-1}\right)\propto \sum_{\theta \in \Theta }1_{\Theta (L(X))}(\theta )\prod_{(x,\alpha ,l)\in X_{k-1}}\prod_{v=1}^{V}\psi_{Z_{k-1}^{\left(v\right)}}^{\left(\theta^{\left(v\right)}(l)\right)}\left(x,\;\alpha_{v},\;l\right).$$

### 4.2. Adaptive CPHD Filter

Within the framework of multi-target tracking, the CPHD filter, based on the independent and identically distributed (i.i.d.) cluster process, assumes that each element in the multi-target cluster follows an i.i.d. distribution and allows for arbitrary cardinality distributions. Although this method cannot provide specific trajectory identity information, the CPHD filter is computationally efficient because it simplifies the association problem between trajectories and measurements [23]. Therefore, this paper embeds the CPHD filter into the MS-GLMB filter to achieve real-time online estimation of the clutter rate and detection probability.

The closed-form solution of this filter is obtained by constructing an augmented mixed state space, which consists of the true target state space and the clutter-generated target state space, with the detection probability treated as part of the state space. Specifically, let the state spaces for the actual target, clutter target, and detection probability be denoted as $X^{\left(1\right)}$, $X^{\left(0\right)}$, and $X^{\left(\Delta \right)}=[0,\;1]$, respectively; then, the mixed state space is

(36) $$X^{\left(h\right)}=\left(X^{\left(1\right)}\times X^{\left(\Delta \right)}\right)⊎\left(X^{\left(0\right)}\times X^{\left(\Delta \right)}\right).$$

To facilitate the description of the recursive computation of PHD and the consistency distribution in the CPHD filter, we establish the following dynamic and measurement models within the aforementioned mixed state space. The state of a single true target is defined as $x_{r}^{\left(h\right)}=\left(x_{r},\;a\right)\in X^{\left(1\right)}\times X^{\left(\Delta \right)}$, where $a\in X^{\left(\Delta \right)}$ is its detection probability. The state of a single clutter target is defined as $x_{c}^{\left(h\right)}=\left(x_{c},\;b\right)\in X^{\left(0\right)}\times X^{\left(\Delta \right)}$, where $b\in X^{\left(\Delta \right)}$ is its detection probability. Based on the statistical independence between true targets and clutter targets, for sensor $v^{th}$, the integral of the function $f^{\left(h\right)}:X^{\left(h\right)}\to R$ can be written as

(37) $$\int_{X^{\left(h\right)}}f^{\left(v,h\right)}\left(x^{\left(h\right)}\right)dx^{\left(h\right)}=\int_{X^{\left(\Delta \right)}}\int_{X^{\left(1\right)}}f^{\left(v,h\right)}\left(x_{r},\;a\right)dx_{r}da+\int_{X^{\left(\Delta \right)}}\int_{X^{\left(0\right)}}f^{\left(v,h\right)}\left(x_{c},\;b\right)dx_{c}db.$$

The joint single-target survival probability is defined as follows

(38) $$p_{S_{k}}^{\left(v,h\right)}\left(x^{\left(h\right)}\right)=\left\{\begin{array}{l} p_{S_{k}}^{\left(1\right)},\;\;\mathrm{if}\;\;\;x^{\left(h\right)}\in X^{\left(1\right)}\times X^{\left(\Delta \right)}\\ p_{S_{k}}^{\left(0\right)},\;\;\mathrm{if}\;\;\;x^{\left(h\right)}\in X^{\left(0\right)}\times X^{\left(\Delta \right)} \end{array}\right..$$

The joint transition density is defined as

(39) $$f_{k}^{\left(v,h\right)}\left(x_{k}^{\left(h\right)}|{x_{k-1}}^{\left(h\right)}\right)=\left\{\begin{array}{ll} f_{k}^{\left(1\right)}\left(x_{r_{k}}|x_{r}\right)f_{k}\left(a_{k}|a\right), & \;\mathrm{if}\;x_{k}^{\left(h\right)}=\left(x_{r_{k}},\;a_{k}\right)\;\mathrm{and}\;{x_{k-1}}^{\left(h\right)}=\left(x_{r},\;a\right)\in X^{\left(1\right)}\times X^{\left(\Delta \right)}\\ f_{k}^{\left(0\right)}\left(x_{c_{k}}|x_{c}\right), & \;\mathrm{if}\;x_{k}^{\left(h\right)}=\left(x_{c_{k}},\;b_{k}\right)\mathrm{and}\;{x_{k-1}}^{\left(h\right)}=\left(x_{c},\;b\right)\in X^{\left(0\right)}\times X^{\left(\Delta \right)}\\ 0, & \;\mathrm{otherwise} \end{array}\right..$$

The joint birth rate at the next time step is defined as

(40) $$\gamma_{k}^{\left(v,h\right)}\left(x_{k}^{\left(h\right)}\right)=\left\{\begin{array}{ll} \gamma_{k}^{\left(1\right)}\left(x_{r_{k}},\;a_{k}\right), & \mathrm{if}\;\;x_{k}^{\left(h\right)}=\left(x_{r_{k}},\;a_{k}\right)\in X_{k}^{\left(1\right)}\times X_{k}^{\left(\Delta \right)}\\ \gamma_{k}^{\left(0\right)}\left(x_{c_{k}},\;b\right), & \mathrm{if}\;\;x_{k}^{\left(h\right)}=\left(x_{c_{k}},\;b\right)\in X_{k}^{\left(0\right)}\times X_{k}^{\left(\Delta \right)} \end{array}\right..$$

The joint birth rate distribution is defined by convolution as follows

(41) $$\rho_{X_{B_{k}}}^{\left(v,h\right)}\left(n^{\left(h\right)}\right)=\left(\rho_{X_{B_{k}}}^{\left(1\right)}*\rho_{X_{B_{k}}}^{\left(0\right)}\right)\left(n^{\left(h\right)}\right).$$

The joint detection probability is defined as

(42) $$p_{D}^{\left(v,h\right)}\left(x^{\left(h\right)}\right)=\left\{\begin{array}{lll} a, & \;\mathrm{if}\; & x^{\left(h\right)}=\left(x_{r},\;a\right)\in X^{\left(1\right)}\times X^{\left(\Delta \right)}\\ b, & \;\mathrm{if}\; & x^{\left(h\right)}=\left(x_{c},\;b\right)\in X^{\left(0\right)}\times X^{\left(\Delta \right)} \end{array}\right..$$

The joint measurement likelihood function is defined as

(43) $$g^{\left(v,h\right)}\left(z|x^{\left(h\right)}\right)=\left\{\begin{array}{ll} g^{\left(1\right)}\left(z|x^{\left(h\right)}\right), & \;\mathrm{if}\;\;x^{\left(h\right)}=\left(x_{r},\;a\right)\in X^{\left(1\right)}\times X^{\left(\Delta \right)}\\ g^{\left(0\right)}(z), & \;\mathrm{if}\;\;x^{\left(h\right)}=\left(x_{c},\;b\right)\in X^{\left(0\right)}\times X^{\left(\Delta \right)} \end{array}\right..$$

Different from existing single-sensor adaptive CPHD filters that bootstrap their own unlabeled PHD prior, here we extract the PHD and cardinality distribution directly from the labeled GLMB posterior. This design avoids maintaining two statistically inconsistent representations of the multi-target state.

### 4.3. Estimation of the Detection Probability and the Noise Rate

The sensor $v^{th}$ runs an independent adaptive CPHD filter; we use the posterior density from the previous time step provided by the MS-GLMB filter as prior information, rather than the prior information from the adaptive CPHD filter itself. Since the MS-GLMB filter fuses measurements from all V sensors, the target information it contains is more accurate than that from a single-sensor adaptive CPHD filter. We use the posterior GLMB density from the previous time step to compute the PHD and cardinality distributions for the current time step, ignoring the target labels themselves and extracting only their overall statistical characteristics. From the GLMB posterior density in (13), the PHD and cardinality distributions of the true target are calculated as

(44) $${\tilde{\zeta }}^{\left(1\right)}\left(x\right)=\sum_{I,\xi }\sum_{l\in I}\omega^{\left(I,\xi \right)}p^{\left(\xi \right)}(x,l)p^{\left(\xi \right)}\left(\alpha_{v}\right)\prod_{i\in \{1:V\}-\{v\}}\int p^{\left(\xi \right)}\left(\alpha_{i}\right)d\alpha_{i},$$

(45) $${\tilde{\rho }}^{\left(1\right)}(n)=\sum_{I,\xi }\delta_{n}(|I|)\omega^{\left(I,\xi \right)},$$

where the cardinality distribution of the target in the mixed state space is ${\tilde{\rho }}^{\left(h\right)}={\tilde{\rho }}^{\left(1\right)}*\rho^{\left(0\right)}$.

Given (44) and (45), and prior information about the current sensor clutter target, the PHD prediction for the true target at the current time step is

(46) $$\zeta_{k}^{\left(v,1\right)}\left(x_{k},\;a_{k}\right)=\gamma^{\left(1\right)}\left(x_{k},\;a_{k}\right)+\iint p_{S}^{\left(1\right)}(x)f_{k}^{\left(1\right)}\left(x_{k}|x\right)f_{\Delta_{k}}^{\left(v\right)}\left(a_{k}|\alpha_{v}\right){\tilde{\zeta }}^{\left(1\right)}\left(x,\;\alpha_{v}\right)d\alpha_{v}dx.$$

The PHD prediction for the clutter target is

(47) $$\zeta_{k}^{\left(v,0\right)}(b)=\gamma^{\left(v,0\right)}(b)+p_{S}^{\left(v,0\right)}\zeta^{\left(v,0\right)}(b).$$

The cardinality distribution prediction for the mixed state space (true targets and clutter targets) is

(48) $$\rho_{k}^{\left(v,h\right)}(n)=\sum_{j=0}^{n}\rho_{k}^{\left(v,h\right)}(n-j)\sum_{i=j}^{\infty }C_{j}^{i}{\tilde{\rho }}^{\left(h\right)}(i){\left(1-f_{v}\right)}^{i-j}\phi_{v}^{j}$$

where $C_{j}^{i}$ is the binomial coefficient, i.e., $C_{j}^{i}=\frac{i!}{j!(i-j)!}$.

The average survival probability of targets in the mixed state space is

(49) $$\phi_{v}=\frac{\langle {\tilde{\zeta }}^{\left(1\right)},\;p_{S}^{\left(1\right)}\rangle +\langle \zeta^{\left(v,0\right)},\;p_{S}^{\left(v,0\right)}\rangle }{\langle 1,\;{\tilde{\zeta }}^{\left(1\right)}\rangle +\langle 1,\;\zeta^{\left(v,0\right)}\rangle }.$$

Given the measurement set $Z_{k}^{\left(v\right)}$, the updated PHD is expressed as

(50) $$\begin{array}{l} \zeta_{k}^{\left(v,1\right)}\left(x_{k},\;a_{k}|Z_{k}^{\left(v\right)}\right)\\ =\zeta_{k}^{\left(v,1\right)}\left(x_{k},\;a_{k}\right)\times \left[\frac{\left(1-a_{k}\right)\times \frac{\langle \Gamma_{k}^{\left(v,1\right)}\left[\zeta_{k}^{\left(v,h\right)},\;Z_{k}^{\left(v\right)}\right],\;\rho_{k}^{\left(v,h\right)}\rangle }{\langle \Gamma_{k}^{\left(v,0\right)}\left[\zeta_{k}^{\left(v,h\right)},\;Z_{k}^{\left(v\right)}\right],\;\rho_{k}^{\left(v,h\right)}\rangle }}{\langle 1,\;\zeta_{k}^{\left(v,1\right)}\rangle +\langle 1,\;\zeta_{k}^{\left(v,0\right)}\rangle }+\sum_{z\in Z_{k}^{\left(v\right)}}\frac{a_{k}\times g^{\left(v,1\right)}\left(z|x_{k}\right)}{\langle \zeta_{k}^{\left(v,0\right)},\;p_{D_{k}}^{\left(v,0\right)}\mu^{\left(v\right)}\rangle +\langle \zeta_{k}^{\left(v,1\right)},\;p_{D_{k}}^{\left(v,1\right)}g^{\left(v,1\right)}(z|\cdot )\rangle }\right], \end{array}$$

(51) $$\begin{array}{l} \zeta_{k}^{\left(v,0\right)}\left(b_{k}|Z_{k}^{\left(v\right)}\right)\\ =\zeta_{k}^{\left(v,0\right)}\left(b_{k}\right)\times \left[\frac{\left(1-b_{k}\right)\times \frac{\langle \Gamma_{k}^{\left(v,1\right)}\left[\zeta_{k}^{\left(v,h\right)},\;Z_{k}^{\left(v\right)}\right],\;\rho_{k}^{\left(v,h\right)}\rangle }{\langle \Gamma_{k}^{\left(v,0\right)}\left[\zeta_{k}^{\left(v,h\right)},\;Z_{k}^{\left(v\right)}\right],\;\rho_{k}^{\left(v,h\right)}\rangle }}{\langle 1,\;\zeta_{k}^{\left(v,1\right)}\rangle +\langle 1,\;\zeta_{k}^{\left(v,0\right)}\rangle }+\sum_{z\in Z_{k}^{\left(s\right)}}\frac{b_{k}\times \mu^{\left(v\right)}(z)}{\langle \zeta_{k}^{\left(v,0\right)},\;p_{D_{k}}^{\left(v,0\right)}\mu^{\left(v\right)}\rangle +\langle \zeta_{k}^{\left(v,1\right)},\;p_{D_{k}}^{\left(v,1\right)}g^{\left(v,1\right)}(z|\cdot )\rangle }\right]. \end{array}$$

The cardinality distribution of the updated mixture state space is

(52) $$\rho_{k}^{\left(v,h\right)}(n)=\left\{\begin{array}{ll} 0, & n<\left|Z_{k}^{\left(v\right)}\right|\\ \frac{\rho^{(n)\cdot 1^{\left(v,0\right)}}\left[\zeta_{k},\;Z_{k}^{\left(v\right)}\right](n)}{\langle \zeta_{k}^{\left(v,h\right)},\;\Gamma_{k}^{\left(v,0\right)}\rangle }, & n\geq \left|Z_{k}^{\left(v\right)}\right| \end{array}\right.$$

where

(53) $$\Gamma_{k}^{\left(v,u\right)}\left[\zeta_{k}^{\left(v,h\right)},\;Z_{k}^{\left(v\right)}\right](n)=\left\{\begin{array}{ll} 0, & n<\left|Z_{k}^{\left(v\right)}\right|+u\\ P_{\left|Z_{k}^{\left(v\right)}\right|+u}^{\left(n\right)}\Phi_{k}^{\left(n-(\left|Z_{k}^{\left(v\right)}\right|+u)\right)}, & n\geq \left|Z_{k}^{\left(v\right)}\right|+u \end{array}\right.,$$

(54) $$\Phi_{k}=1-\frac{\langle \zeta_{k}^{\left(v,1\right)},\;p_{D_{k}}^{\left(v,1\right)}\rangle +\langle \zeta_{k}^{\left(v,0\right)},\;p_{D_{k}}^{\left(v,0\right)}\rangle }{\langle 1,\;\zeta_{k}^{\left(v,1\right)}\rangle +\langle 1,\;\zeta_{k}^{\left(v,0\right)}\rangle },$$

(55) $$p_{D_{k}}^{\left(v,1\right)}(x,a)=a,$$

(56) $$p_{D_{k}}^{\left(v,0\right)}(b)=b,$$

where $P_{j}^{n}$ is the permutation coefficient, i.e., $P_{j}^{n}=\frac{n!}{(n-j)!}$.

The motion state of the target is modeled using a Gaussian distribution, while its detection probability is described by a Beta distribution [23]. The PHD of the clutter target is represented as a mixture of multiple Beta distributions, that is,

(57) $$\zeta^{\left(v,0\right)}\left(b_{k}\right)=\sum_{i=1}^{J^{\left(v,0\right)}}w_{i}^{\left(v,0\right)}\beta_{i}^{\left(v,0\right)}\left(b_{k}|s_{i}^{\left(v,0\right)},\;t_{i}^{\left(v,0\right)}\right)$$

where β(⋅|s,t) denotes a Beta distribution with mean $\frac{s}{\left(s+t\right)}$ and variance $\frac{st}{{\left(s+t\right)}^{2}(s+t+1)}$. The clutter ratio of the sensor $v^{th}$ is calculated as

(58) $${\lambda_{c}}^{\left(v\right)}=\sum_{i=1}^{J^{\left(v,0\right)}}w_{i}^{\left(v,0\right)}\frac{s_{i}^{\left(v,0\right)}}{s_{i}^{\left(v,0\right)}+t_{i}^{\left(v,0\right)}}.$$

In the actual algorithm implementation, the proposed clutter rate estimation module can be designed as a parallel processing architecture to process data from multiple sensors simultaneously.

### 4.4. MS-GLMB Filter State Estimation

After the adaptive CPHD provides a set of clutter rates ${\lambda_{c}}^{\left(1:V\right)}$, the MS-GLMB filter estimates the mixed states of the cluster targets [34]. Considering the detection probability of multi-sensor targets a, and using the GLMB density from (13), the updated GLMB density can be expressed as

(59) $$\begin{array}{c} \pi_{k|k}\left(X_{k}\right)\propto \Delta \left(X_{k}\right)\sum_{I,\xi ,I_{k}}\omega_{k}^{\left(I,\xi ,I_{k}\right)}\delta_{I_{k}}\left(L\left(X_{k}\right)\right)\sum_{\theta_{k}\in \Theta_{k}}1_{\Theta_{k}\left(L\left(X_{k}\right)\right)}\left(\theta_{k}\right){\left[\Psi_{Z}^{\left(\theta_{-}(\cdot )\right)}(\cdot )p_{k}^{\left(\xi \right)}(\cdot )\right]}^{X_{k}}\\ \propto \Delta \left(X_{k}\right)\sum_{I,\xi ,I_{k},\theta_{k}}\omega_{k}^{\left(I,\xi ,I_{k}\right)}\omega_{Z}^{\left(I,\xi ,I_{k},\theta_{k}\right)}\delta_{I_{k}}\left(L\left(X_{k}\right)\right){\left[p_{Z}^{\left(\xi ,\theta_{k}\right)}\right]}^{X_{k},} \end{array}$$

where

(60) $$\omega_{Z}^{\left(I,\xi ,I_{k},\theta_{k}\right)}=1_{\Theta_{k}\left(I_{k}\right)}\left(\theta_{k}\right){\left[1-{\bar{P}}_{S}^{\left(\xi \right)}\right]}^{I-I_{k}}{\left[{\bar{P}}_{S}^{\left(\xi \right)}\right]}^{I\cap I_{k}}{\left[1-r_{B,k}\right]}^{B_{k}-I_{k}}r_{B,k}^{B_{k}\cap I_{k}}{\left[{\bar{\psi }}_{Z}^{\left(\xi ,\theta_{k}\right)}\right]}^{I_{k}},$$

(61) $${\bar{P}}_{S}^{\left(\xi \right)}(l)=\langle p^{\left(\xi \right)}(\cdot ,l),P_{S}(\cdot ,l)\rangle ,$$

(62) $${\bar{\psi }}_{Z}^{\left(\xi ,\theta_{k}\right)}\left(l_{k}\right)=\int {\bar{p}}_{k}^{\left(\xi \right)}\left(x_{k},\;l_{k}\right)\prod_{v=1}^{V}p^{\left(\xi \right)}\left(\alpha_{v}\right)\psi_{Z}^{\left(\theta_{k}\left(l_{k}\right)\right)}\left(x_{k},\;\alpha_{k},\;l_{k}\right)dx_{k}d\alpha_{1:V},$$

(63) $$p_{S}\left(x_{k},\;\alpha_{k},\;l_{k}\right)=\int P_{S}\left(x,\;l_{k}\right)f_{S_{k}}\left(x_{k}|x,\;l_{k}\right)p^{\left(\xi \right)}\left(x,\;l_{k}\right)dx\times \prod_{v=1}^{V}\int p^{\left(\xi \right)}\left(\alpha_{v}\right)f_{\Delta_{k}}^{\left(v\right)}\left(\alpha_{v_{k}}|\alpha_{v}\right)d\alpha_{v},$$

(64) $${\bar{p}}_{k}^{\left(\xi \right)}\left(x_{k},\;\alpha_{k},\;l_{k}\right)=1_{L}\left(l_{k}\right)\frac{p_{S}\left(x_{k},\;\alpha_{k},\;l_{k}\right)}{{\bar{P}}_{S}^{\left(\xi \right)}\left(l_{k}\right)}+1_{B_{k}}\left(l_{k}\right)p_{B,k}\left(x_{k},\;l_{k}\right),$$

(65) $$p_{Z}^{\left(\xi ,\theta_{k}\right)}\left(x_{k},\;\alpha_{k},\;l_{k}\right)=\frac{{\bar{p}}_{k}^{\left(\xi \right)}\left(x_{k},\;\alpha_{k},\;l_{k}\right)\psi_{Z_{k}}^{\left(\theta_{k}\left(l_{k}\right)\right)}\left(x_{k},\;\alpha_{k},\;l_{k}\right)}{{\bar{\psi }}_{Z}^{\left(\xi ,\theta_{k}\right)}\left(l_{k}\right)},$$

### 4.5. Multi-Sensor Measurement Partitioning

In the MS-GLMB filtering framework, the core of the update step is to associate the joint measurement set $Z=(Z^{\left(1\right)},\ldots ,Z^{\left(V\right)})$ with the predicted latent target set, i.e., measurement partitioning. Traditional iterative estimator methods circumvent this challenge by processing the measurement sets of each sensor individually; however, their performance is highly dependent on the sensor processing order, and critical assumptions may be lost during each iteration, leading to suboptimal filtering. To address this issue, this paper adopts a joint prediction and update strategy that uniformly models the multi-sensor data association problem as a multi-dimensional assignment problem. However, when the number of sensors V>2, this problem is NP-hard, and direct solution is computationally infeasible.

To efficiently solve this multidimensional assignment problem, this paper draws on a truncation strategy based on Gibbs sampling [35]. This method does not seek a global optimal solution but efficiently generates a set of high-weight association hypotheses by randomly sampling from the posterior distribution of association hypotheses. First, to uniformly describe the target’s survival/reappearance and its association with measurements from each sensor, an extended association mapping γ is introduced. For a system comprising P latent targets (from the survival set I and the newborn set $B_{+}$) and V sensors, γ can be viewed as a P×V array. Its *n*th row $\gamma_{n}=(\gamma_{n}^{\left(1\right)},\ldots ,\gamma_{n}^{\left(V\right)})$ describes the association status of the *n*th target $l_{n}$. If target $l_{n}$ has died or not been born, then $\gamma_{n}=(-1,\ldots ,-1)$. If target $l_{n}$ is alive or has been born, then $\gamma_{n}^{\left(v\right)}\in \{0,1,\ldots ,M^{\left(V\right)}\}$, where 0 indicates a missed detection by sensor v, and $j\in \{1,\ldots ,M^{\left(V\right)}\}$ indicates an association with the *j*th measurement of sensor v.

Given an association map γ, whose weight in the GLMB update is proportional to the posterior probability $\pi (\gamma |Z_{k})$ [35], this probability can be decomposed as

(66) $$\pi (\gamma |Z_{k})\propto 1_{\Gamma }(\gamma )\prod_{n=1}^{P}\eta_{n}(\gamma_{n}),$$

where $1_{\Gamma }(\gamma )$ is an indicator function ensuring that each measurement is assigned to at most one target (i.e., the validity of the association mapping). $\eta_{n}(\gamma_{n})$ is the weighting factor associated with the *n*th target, which combines the target’s survival/new onset probability, detection probability, and the likelihood associated with the corresponding measurement $z_{\gamma_{n}}$ [36].

Gibbs sampling simulates the entire joint distribution by iteratively sampling from the conditional probability distribution of each variable. Here, this involves sequentially sampling the association mapping $\gamma_{n}$ for each target $l_{n}$, whose conditional distribution is $p(\gamma_{n}|\gamma_{\bar{n}},Z_{k})$, where $\gamma_{\bar{n}}$ represents the association mappings of all other targets except $l_{n}$.

The computational complexity of sampling directly from this conditional distribution is proportional to the number of all possible association states, i.e., $O(\prod_{v=1}^{V}\left(M^{\left(v\right)}+1\right))$. As the number of sensors and measurements increases, this computational load becomes enormous. To achieve algorithmic scalability, a suboptimal minimum Markov approximation strategy [37] is adopted. Specifically, given a target’s state, the detection events of that target across different sensors are assumed to be independent of one another. Based on this assumption, the joint likelihood is decomposed into the product of individual likelihoods

(67) $${\bar{\psi }}_{Z}^{\left(\gamma_{n}\right)}(l_{n})\approx \prod_{v=1}^{V}{\bar{\psi }}_{Z}^{\left(v,\gamma_{n}^{\left(v\right)}\right)}(l_{n}).$$

This approximation allows $\eta_{n}(\gamma_{n})$ to be decomposed into the form $\prod_{v=1}^{V}\eta_{n}^{\left(v\right)}(\gamma_{n}^{\left(v\right)})$. Consequently, the sampling of the multidimensional vector $\gamma_{n}$ can be decomposed into independent sequential sampling of each component $\gamma_{n}^{\left(v\right)}$. This significantly reduces the computational complexity of the sampling process from a product of the number of measurements from each sensor to a sum

(68) $$O\left(\prod_{v=1}^{V}M^{\left(v\right)}\right)\to O\left(\sum_{v=1}^{V}M^{\left(v\right)}\right).$$

This reduction in complexity enables the method to be effectively applied to scenarios with a large number of sensors, and its performance is independent of the sensor processing order.

The overall computational complexity of one filtering cycle of MS-AGLMB is $O(K\cdot N_{max}\cdot M_{max}\cdot r+C\cdot G\cdot logG)$, where *K* is the number of sensors, $N_{max}$ is the maximum number of targets, $M_{max}$ Mmax is the maximum number of measurements per sensor, *r* is the number of retained components, *C* is the complexity of the CPHD prediction step, and *G* is the number of Gibbs samples. Due to the suboptimal Markov approximation (Equation (67)), the Gibbs sampling cost is proportional to the sum of the measurements across sensors, rather than their product. The adaptive CPHD estimator introduces a linear term in both the sensor count and the number of particles, which is typically smaller than the GLMB update cost.

### 4.6. State Extraction and Cluster Structure Estimation

After each filtering update, the GLMB posterior density contains state information for all true and clutter targets. To generate prior information usable for the next time step, state extraction and cluster structure estimation must be performed.

Given the posterior GLMB density $\pi_{k}(X)$ at time k, we iterate over all posterior hypotheses. For each label $l\in L_{k}$, we compute its marginal presence probability $r_{k}^{\left(l\right)}$ and the corresponding state probability density function $p_{k}^{\left(l\right)}(x)$. The presence probability is obtained by summing the weights of all hypotheses containing that label

(69) $$r_{k}^{\left(l\right)}=\sum_{(L,\theta )\in F\left(L_{k}\right)\times \Theta }w^{\left(L,\theta \right)}\cdot 1_{L}(l).$$

For labels with a presence probability exceeding the preset threshold $\tau_{\mathrm{exist}\;}$, the corresponding target is considered to be present. In the Gaussian mixture implementation of the generalized label multi-Bernoulli filter, the state density for each label l can be expressed as

(70) $$p_{k}^{\left(l\right)}(x)=\sum_{j=1}^{J_{k}^{\left(l\right)}}\omega_{j,k}^{\left(l\right)}N\left(x;\;m_{j,k}^{\left(l\right)},\;P_{j,k}^{\left(l\right)}\right),$$

where $J_{k}^{\left(l\right)}$ is the number of Gaussian components corresponding to the label, and $\omega_{j,k}^{\left(l\right)}$, $m_{j,k}^{\left(l\right)}$, and $P_{j,k}^{\left(l\right)}$ represent the weight, mean, and covariance matrix of the *j*th Gaussian component, respectively. By computing the population mean and covariance of this mixture density, a point estimate of the target and a measure of its uncertainty can be obtained. The population state estimate is

(71) $${\hat{x}}_{k}^{\left(l\right)}=\sum_{j=1}^{J_{k}^{\left(l\right)}}\omega_{j,k}^{\left(l\right)}m_{j,k}^{\left(l\right)}$$

Accordingly, the calculation of the overall covariance matrix must simultaneously account for the covariances of each Gaussian component and the dispersion of the component means relative to the overall mean.

(72) $$P_{k}^{\left(l\right)}=\sum_{j=1}^{J_{k}^{\left(l\right)}}\omega_{j,k}^{\left(l\right)}\left[P_{j,k}^{\left(l\right)}+\left(m_{j,k}^{\left(l\right)}-{\hat{x}}_{k}^{\left(l\right)}\right){\left(m_{j,k}^{\left(l\right)}-{\hat{x}}_{k}^{\left(l\right)}\right)}^{T}\right],$$

Thus, the set of multi-target state estimates at time k is obtained

(73) $${\hat{X}}_{k}=\left\{\left({\hat{x}}_{k}^{\left(l\right)},\;P_{k}^{\left(l\right)},\;l\right):\;l\in L_{k},\;r_{k}^{\left(l\right)}>\tau_{\mathrm{exist}\;}\right\}.$$

Based on the extracted multi-target state estimates, the cluster structure can be estimated using (19) and (20), and the estimated cluster structure information is fed back into the prediction step for the next time step.

## 5. Experiments and Analysis

To evaluate the tracking performance of the proposed MS-AGLMB filter for cluster targets, we set up three simulation scenarios to assess the filter’s tracking accuracy, its adaptive capabilities regarding clutter ratio and detection probability, and the contributions of each module to tracking accuracy. In the experiments, we simultaneously employed the GLMB filter [18], DP-GLMB filter [36], MS-GLMB filter [37], MS-MeMBer filter [38], and the proposed MS-AGLMB filter to track cluster targets. The capabilities of each filter are shown in Table 1.

In the experimental scenario, the sensor monitoring area is set to [0, 2000] × [0, 2000] m^2^. Targets are randomly generated in clusters within four regions, with region centers at ${\left[400,\;400\right]}^{T}\;m$, ${\left[600,\;400\right]}^{T}\;m$, ${\left[600,\;600\right]}^{T}\;m$, and ${\left[400,\;600\right]}^{T}\;m$. The target’s motion state is represented by a four-dimensional vector containing planar position and velocity, i.e., $x_{k}={\left[p_{x,k},\;p_{y,k},\;{\dot{p}}_{x,k},\;{\dot{p}}_{y,k}\right]}^{T}$. The initial states of cluster members follow a Gaussian distribution with a transition density of

(74) $$f_{S_{k}}\left(x_{k}|x,\;l\right)=N\left(x_{k};\;Fx,\;Q\right)$$

where $N(\cdot ;\bar{x},P)$ represents a Gaussian distribution with mean $\bar{x}$ and covariance P, $F_{k}=\left[\begin{array}{cc} I_{2} & \Delta tI_{2}\\ O_{2} & I_{2} \end{array}\right]$, $Q=\sigma_{a}^{2}\left[\begin{array}{cc} \frac{\Delta t^{4}}{4}I_{2} & \frac{\Delta t^{3}}{2}I_{2}\\ \frac{\Delta t^{3}}{2}I_{2} & \Delta t^{2}I_{2} \end{array}\right]$, $\;\sigma_{a}$ is the noise covariance matrix, set to $\;\sigma_{a}=0.1\;m\cdot s^{-1}$.

The target survival probability is set to $p_{s}=0.99$, and new targets follow the LMB model, with a birth probability of $r_{b}=0.05$ at each birth point. The total simulation duration is $N_{T}=100\;s$, and the sampling period is Δt=1 s.

The multi-sensor measurement parameters are set as follows. By default, this experiment configures v=3 sensors located at $s^{\left(v\right)}=\left(s_{x}^{\left(v\right)},\;s_{y}^{\left(v\right)}\right)$, specifically ${\left[500,\;700\right]}^{T}\;m$, ${\left[800,\;300\right]}^{T}\;m$, and ${\left[200,\;300\right]}^{T}\;m$. The sensor observation model in this experiment adopts a nonlinear polar coordinate model, defined by the observation vector of sensor v at time k as given by (28)

(75) $$z_{v,k}=\left[\frac{\mathrm{atan}2\left(p_{x,k}-s_{x}^{\left(v\right)},\;p_{y,k}-s_{y}^{\left(v\right)}\right)}{\sqrt{{\left(p_{x,k}-s_{x}^{\left(v\right)}\right)}^{2}+{\left(p_{y,k}-s_{y}^{\left(v\right)}\right)}^{2}}}\right]+v_{k}.$$

Measurement noise $v_{k}\sim N\left(0,\;R_{j}\right)$, with covariance matrix $R_{j}=\mathrm{diag}\left({\left[\sigma_{\theta }^{2},\;\sigma_{r}^{2}\right]}^{T}\right)$, where the standard deviation of angular error is $\sigma_{\theta }=1.0{}^{\circ}$ and the standard deviation of range error is $\sigma_{r}=5\;m$. The field of view (FOV) of each sensor covers an azimuth range of [−π/2, π/2] radians and a range of [0, 2000] m. Clutter follows a uniform distribution within the FOV. The detection probability $p_{i,D}$ and average clutter rate $\lambda_{c}$ for each scenario are set as shown in Table 2. In Scenario 1, we set a low detection probability and a high average clutter rate to simulate an unknown detection environment, in order to evaluate the algorithm’s tracking capability; in Scenario 2, we set varying detection probabilities and clutter rates to simulate a changing detection environment, in order to evaluate the algorithm’s adaptability. Clutter is uniformly distributed within the sensor’s field of view V=[−π/2, π/2]×[0, 2000], with a corresponding spatial density of clutter $c(\cdot )=\lambda_{C}/|V|$. The number of sensors for the Single-GLMB filter is v=1, while the number of sensors used by other filters is v=3. In this experiment, for the multi-sensor system with the default configuration v=3, the detection space is much larger than the coverage range of a single sensor, thereby increasing the complexity of data association.

The filter parameters are set as follows. The maximum number of retained hypotheses for the MS-AGLMB filter is $H_{\max\;}=3000$, and the pruning threshold is $\Gamma =10^{-10}$. A nonlinear model is handled using unscented Kalman filter (UKF) [39], with the scale parameter set to α=1, β=2, κ=2. The elliptical gate probability is set to $P_{G}=0.99$, and the corresponding Mahalanobis distance threshold $G_{m}$ is determined by the chi-squared distribution $\chi_{2}^{2}\left(P_{G}\right)$ under the observation dimension. The detection probability $p_{j,D}$ is modeled as a Beta distribution $p_{j,D}\sim \mathrm{Beta}(\alpha ,\;\beta )$, with the prior initialized as Beta (18, 2) (mean 0.9). The clutter rate is estimated using an adaptive strategy based on a CPHD filter, with a Gaussian component pruning threshold of $\Gamma_{u}=10^{-5}$, a merging threshold of 4, a maximum number of components of $L_{\max}=100$, and an upper bound for the base distribution of $N_{\max}=100$. For the comparison algorithms, the detection probability $p_{i,D}$ and clutter rate $\lambda_{c}$ both use the sensor ground truth, whereas for MS-AGLMB, the detection probability $p_{i,D}$ and average clutter rate $\lambda_{c}$ are initialized with prior values of 0.90 and 5, respectively, which differ from the sensor ground truth.

We used the same parameter values and random noise for each scenario. We conducted 100 Monte Carlo simulations. The estimation error was quantified using the optimal sub-pattern assignment (OSPA) and OSPA^(2)^ metrics [40]. For OSPA, given the true value $X={\left\{x_{i}\right\}}_{i=1}^{n}$. We estimated $Y={\left\{y_{j}\right\}}_{j=1}^{m}$, truncated c>0 with order p≥1,

(76) $$d_{\mathrm{OSPA}\;}^{\left(c,p\right)}(Y,\;X)={\left(\frac{1}{\max(m,n)}\left(c^{p}|m-n|+\min_{\pi }\sum_{i=1}^{\min(m,n)}d^{\left(c\right)}{\left(x_{i},\;y_{\pi (i)}\right)}^{p}\right)\right)}^{1/p}$$

where $d^{\left(c\right)}(x,\;y)=\min(\|x-y\|,\;c)$ and π are optimal permutations. In this experiment, we set c=100 and p=1, and report the total error, localization component, and cardinality component separately. For OSPA^(2)^, given a window length of $w_{l}$,

(77) $${\mathrm{OSPA}}_{l}^{\left(2\right)}={\left(\frac{e^{v}|M-N|+{\mathrm{cost}}_{t}}{\max(M,N)}\right)}^{1/p}$$

where ${\mathrm{cost}}_{l}$ is the matching cost obtained via the Hungarian algorithm from the time-averaged OSPA distance matrix between trajectories within the window, and M and N represent the number of valid trajectories within the window. In this experiment, we use c=100, p=1, and $w_{l}=10$.

### 5.1. Scenario 1

**Figure 3:**
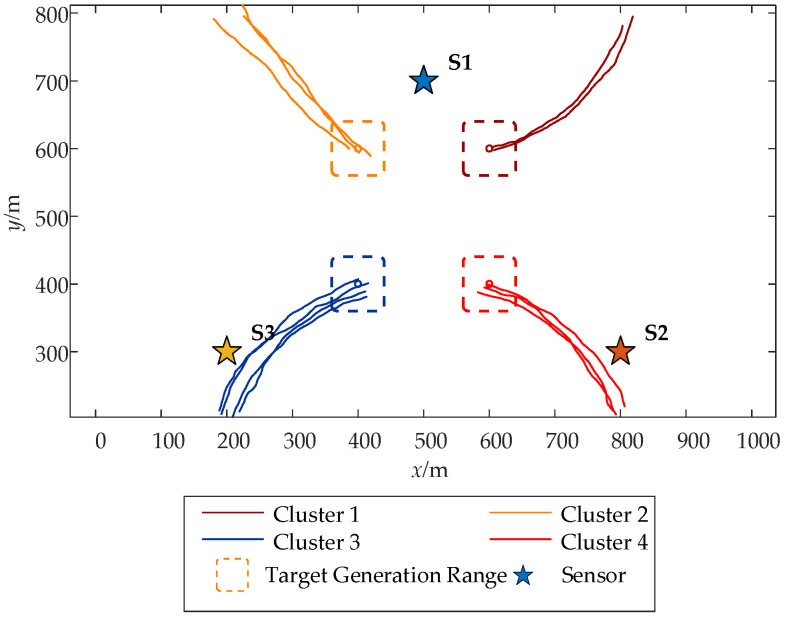
Cluster target trajectory.

Scenario 1 was constructed to test the filter’s accuracy in cluster target state estimation. Four clusters appear at different times: cluster $C_{1}$ (2 members) appears in region $R_{1}$ at k=5 s; cluster $C_{2}$ (3 members) appears in region $R_{2}$ at k=20 s; cluster $C_{3}$ (4 members) appears in region $R_{3}$ at k=20 s; cluster $C_{4}$ (3 members) appears in region $R_{4}$ at k=30 s. Subsequently, the clusters disappeared one after another: cluster $C_{1}$ disappeared at k=75 s, cluster $C_{2}$ disappeared at k=80 s, cluster $C_{3}$ disappeared at k=90 s, and cluster $C_{4}$ disappeared at k=100 s. The trajectories of the aforementioned clusters are shown in Figure 3.

**Figure 4:**
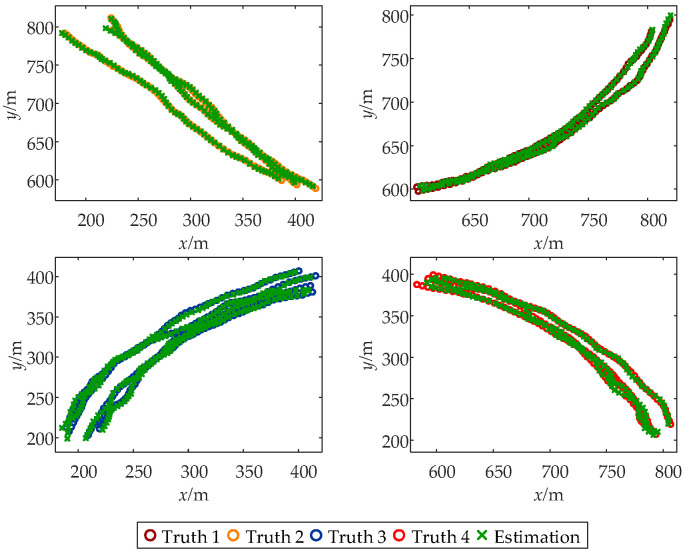
Target tracking results of the MS-AGLMB filter in scenario 1.

As shown in Figure 3, after formation of each cluster, the distances between cluster members gradually diverge from a compact state and eventually stabilize, influenced by both cooperative effects and maneuvering randomness. Figure 4 displays the tracking results of our proposed algorithm. As shown in Figure 4, the MS-AGLMB filter effectively adapts to the aforementioned dynamic cooperative maneuvering process, achieving accurate individual estimation of members within the cluster. It can also accurately distinguish targets when their trajectories intersect.

**Figure 5:**
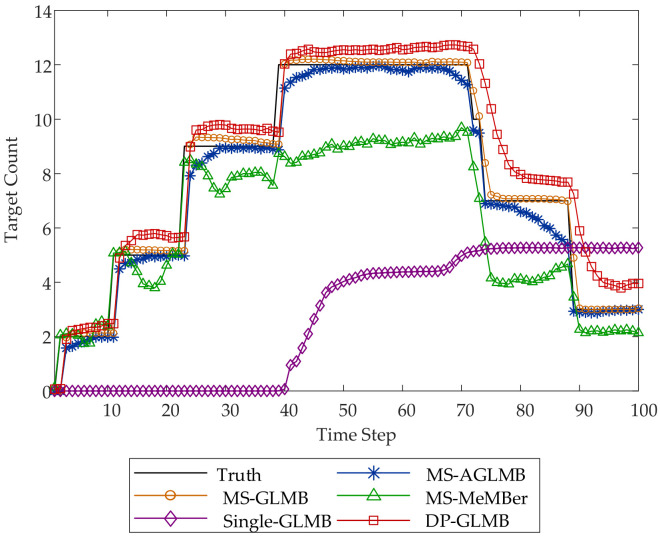
Estimated number of members in scenario 1.

Figure 5 illustrates the estimation and recognition capabilities of each algorithm for clustered targets. As shown in Figure 5, MS-GLMB, DP-GLMB, and MS-AGLMB are capable of accurately estimating clustered targets. The estimation performance of our proposed MS-AGLMB is nearly identical to that of MS-GLMB, which uses true sensor parameters, and is overall superior to DP-GLMB, which also possesses adaptive capabilities. MS-MeMBer exhibits poor tracking performance, while Single-GLMB is virtually unable to track clustered targets. The MeMBer framework cannot output labeled target estimates, making it prone to missed detections and misclassifications when target trajectories intersect or objects are occluded. Single-GLMB relies solely on measurements from a single sensor, making it prone to losing track of targets, whereas MS-AGLMB can comprehensively utilize multi-sensor measurements to achieve better target estimation.

**Figure 6:**
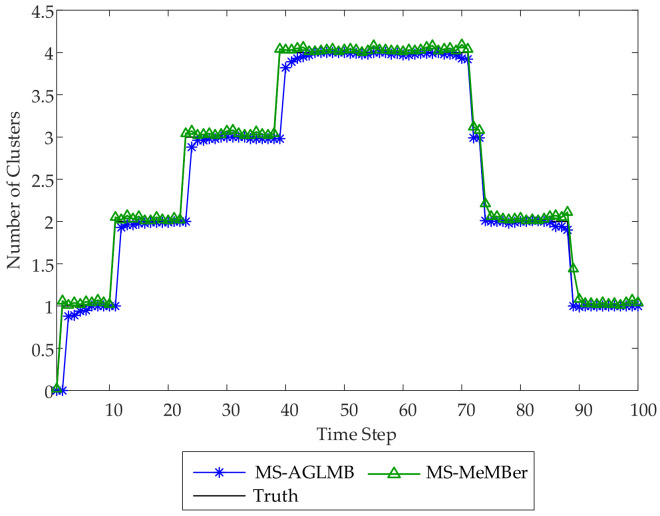
Estimated number of clusters in scenario 1.

Figure 6 demonstrates the cluster tracking capability of our proposed algorithm. As shown in Figure 6, MS-AGLMB exhibits nearly identical cluster tracking performance to the MS-MeMBer filter with real parameters, thanks to the adaptive CPHD filter’s real-time estimation of detection probability and clutter rate.

**Figure 7:**
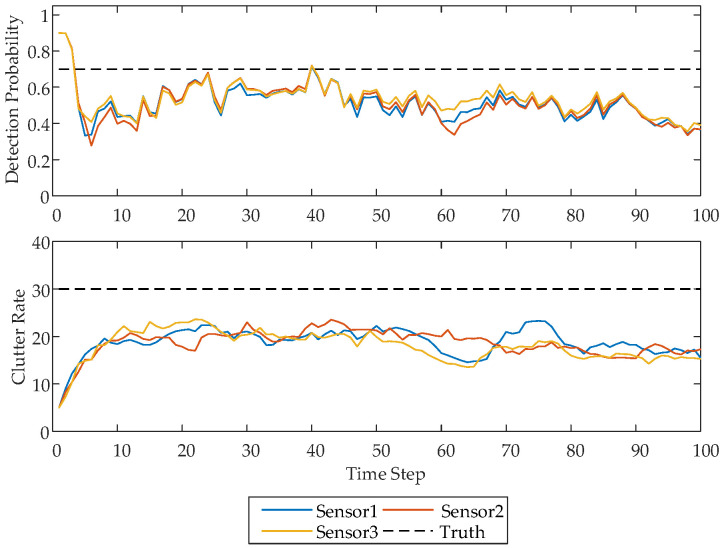
Mean estimated detection probability and mean clutter rate in scenario 1.

Figure 7 demonstrates the adaptive capabilities of our filter regarding detection probability and clutter rate. As shown in Figure 7, the detection probability, starting from an initial value of 0.90, converges to the sensor’s true value of approximately 0.70 through real-time estimation by the adaptive CPHD filter. The clutter rate, starting from an initial value of 5, stabilizes at 20, which is slightly lower than the sensor’s true value. This demonstrates the adaptive estimation capabilities of our algorithm.

**Figure 8:**
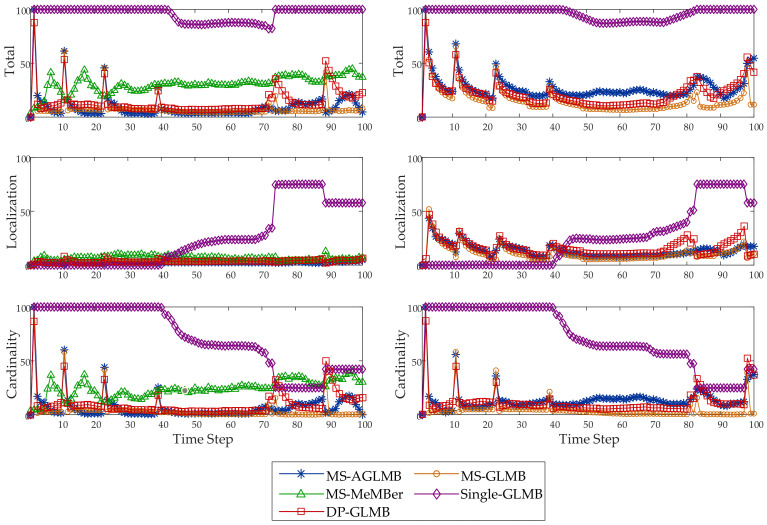
Mean OSPA (**left**) and OSPA^(2)^ (**right**) errors in scenario 1.

Figure 8 presents the OSPA and OSPA^(2)^ errors of each algorithm throughout the tracking process. From Figure 8, it can be seen that MS-AGLMB yields low estimation errors in terms of the overall error, the localization component, and the cardinality component. Although MS-GLMB adopts the true sensor parameters, it does not possess the capability of group structure determination, leading to larger errors when clusters appear or disappear. The DP-GLMB filter similarly exhibits larger errors during the appearance and disappearance of clusters. Even though MS-AGLMB does not have access to the true detection probability and clutter rate, it can still achieve estimation capability close to that of the ideal MS-GLMB when the cluster state changes, and it achieves even smaller errors after the cluster state changes, thanks to the accurate identification of cluster states by the virtual leader–follower group structure model. The estimation error of MS-MeMBer is larger than those of MS-GLMB and MS-AGLMB because it lacks label management capability, while the Single-GLMB incurs large estimation errors due to target loss.

### 5.2. Scenario 2

Scenario 2 was designed to evaluate our algorithm’s adaptability to unknown clutter rates and detection probabilities. The same cluster motion trajectories as in Scenario 1 were used. For the MS-GLMB, MS-MeMBer, DP-GLMB, and Single-GLMB filters, the actual sensor parameters were used, while the initial values for the detection probability and clutter rate of the MS-AGLMB filter were set to 0.90 and 5, respectively. For each sensor, during the first 30 time steps, the detection probability and clutter rate were set to 0.7 and 30, respectively, to simulate a challenging tracking environment; during the subsequent 70 time steps, these values were changed to 0.9 and 5, respectively. This variation in parameters was used to evaluate the adaptability of our proposed algorithm to unknown parameters.

**Figure 9:**
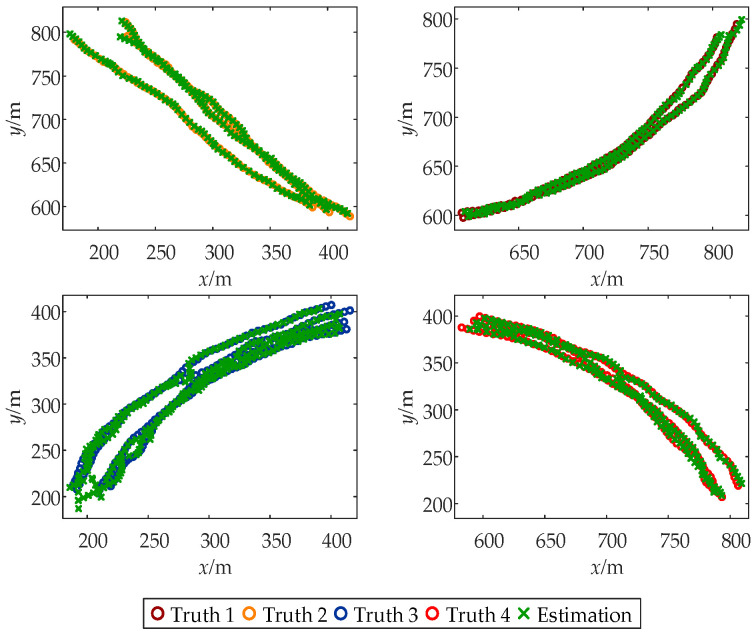
Target tracking results of the MS-AGLMB filter in scenario 2.

Figure 9 illustrates the tracking performance of our proposed algorithm under varying parameter conditions. As shown in Figure 9, the estimation results remain stable during the initial phase of target motion. After the sudden changes in detection probability and clutter rate, there is no significant impact on the estimation results, and the MS-AGLMB filter continues to accurately estimate the target. This demonstrates that our proposed algorithm exhibits good adaptability to variations in detection probability and clutter rate under changing parameter conditions.

**Figure 10:**
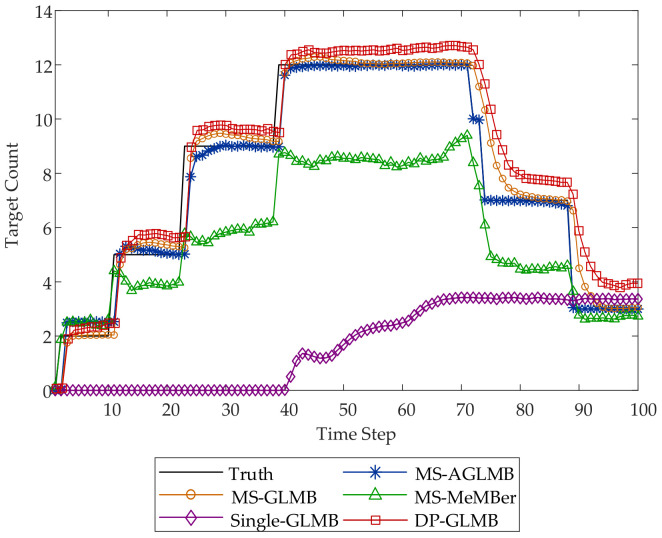
Estimated number of members in scenario 2.

Regarding the cluster target tracking capability, Figure 10 shows that under time-varying parameters, the MS-AGLMB filter still demonstrates tracking performance close to that of the MS-GLMB filter with ideal parameters. It achieves higher tracking accuracy than the DP-GLMB filter and responds more promptly to changes in the number of clusters. Moreover, when the number of targets reaches its peak, the tracking accuracy is superior to those of both MS-GLMB and DP-GLMB, because the group structure model can estimate the targets more accurately, whereas the MS-GLMB is prone to misidentify clutter as targets.

**Figure 11:**
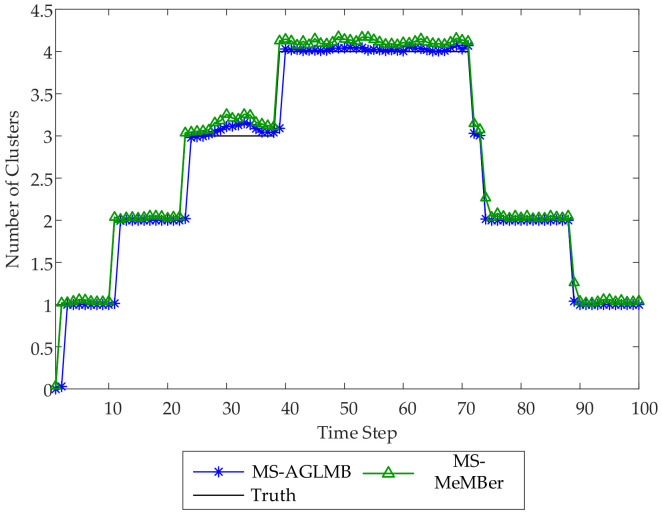
Estimated number of clusters in scenario 2.

As shown in Figure 11, at the 30 s mark, the detection probability and clutter rate undergo a sudden change, causing significant errors in the filters’ estimates of the number of clusters. However, the MS-AGLMB filter still accurately estimates the number of clusters, thanks to the adaptive CPHD filter’s real-time estimation of detection probability and clutter rate. Overall, the MS-AGLMB filter performs better than the MS-MeMBer filter in terms of estimation accuracy.

**Figure 12:**
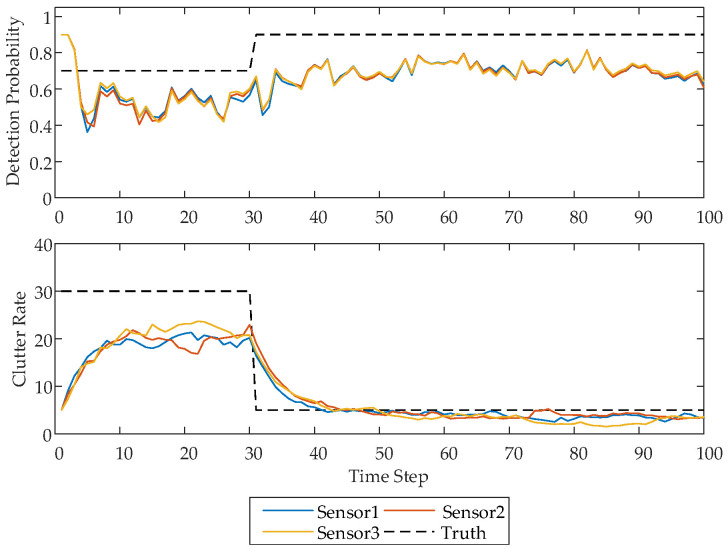
Mean estimated detection probability and mean clutter rate in scenario 2.

As shown in Figure 12, the MS-AGLMB filter correctly estimates the detection probability and clutter rate. While it underestimates the detection probability, its estimation of the clutter rate is both accurate and rapid, which is key to the MS-AGLMB’s ability to accurately estimate the number of clusters. After 30 time steps, the clutter rate stabilizes at the true value of 5.

**Figure 13:**
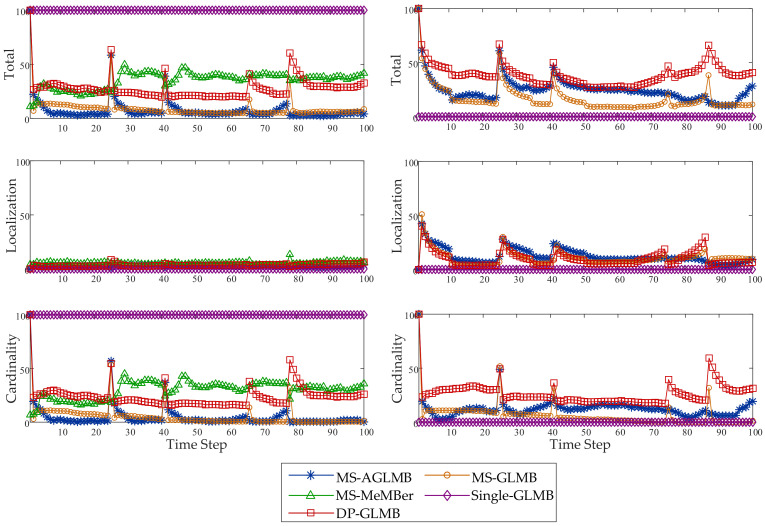
Mean OSPA (**left**) and OSPA^(2)^ (**right**) errors in scenario 2.

From Figure 13, it can be observed that in the early stage of tracking, the errors are noticeable due to the large discrepancy between the initial parameter settings and the true values. However, with the real-time estimation of the adaptive CPHD filter, the errors gradually decrease. The DP-GLMB, which also possesses adaptive capability, exhibits higher tracking errors than our MS-AGLMB. In the later stage of target motion, the errors approach those of the ideal MS-GLMB filter. This is attributed to the precise identification capability of the virtual leader–follower cluster structure model for cluster targets, and it also demonstrates the effectiveness of the adaptive adjustment of detection probability and clutter rate in our algorithm.

In the first scenario, our proposed MS-AGLMB filter achieved tracking performance slightly inferior to that of the ideal-parameter MS-GLMB filter by adjusting the detection probability and clutter rate via the adaptive CPHD filter. In the second scenario, the tracking performance of the MS-AGLMB was generally superior to that of the MS-GLMB with ideal parameters. After changes in sensor parameters, the MS-AGLMB demonstrated even better tracking performance, thanks to the introduction of the virtual leader–follower group structure model. At the same time, we found that under low detection probability conditions, the MS-AGLMB underestimated the clutter rate, while under high detection probability conditions, it was able to accurately estimate the clutter rate.

### 5.3. Scenario 3

Scenario 3 was designed to evaluate the individual contributions of the clustering structure model, adaptive parameter estimation, and Gibbs sampling method in our MS-AGLMB approach. Scenario 3 employs the same cluster motion trajectories as Scenario 1 and the same detection probability and clutter rate parameter settings as Scenario 2, specifically: for the first 30 time steps, the detection probability and clutter rate are 0.7 and 30, respectively; for the subsequent 70 time steps, these values change to 0.9 and 5, respectively. We conducted comparative experiments on five filters, with their functionalities shown in Table 3. MS-GLMB-C disables CPHD estimation, while MS-AGLMB-NC uses only the cluster structure. The initial priors for the detection probability and clutter rate in MS-GLMB-C and MS-AGLMB-NC are 0.9 and 10, respectively. MS-AGLMB-TP uses the true values of the sensor’s detection probability and clutter rate; MS-AGLMB-FP and MS-AGLMB have their detection probabilities and clutter rates fixed at 0.9 and 10, respectively; both MS-AGLMB-TP and MS-AGLMB-FP disable CPHD estimation.

**Figure 14:**
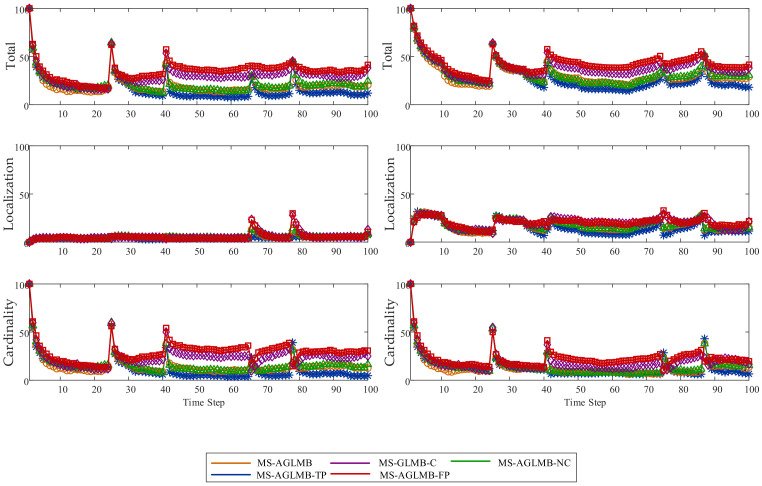
Mean OSPA (**left**) and OSPA^(2)^ (**right**) errors in scenario 3.

As shown in Figure 14, MS-AGLMB-TP (blue), which uses real sensor parameters, achieves the lowest OSPA, while MS-AGLMB-FP (red), which uses fixed-error sensor parameters, achieves the highest OSPA. Since our proposed MS-AGLMB (yellow) does not utilize real sensor parameters, its OSPA is slightly higher than that of MS-AGLMB-TP. Compared to MS-AGLMB-NC (green), MS-AGLMB has a smaller OSPA, thanks to the constraints imposed by the group structure model on cluster motion, particularly in the first 20 time steps. However, we can also observe that the group structure model does not significantly reduce tracking error. Compared to MS-GLMB-C (purple), MS-AGLMB shows a significant reduction in OSPA, particularly after the parameter change at time step 30. This demonstrates the ability of our designed CPHD to improve tracking accuracy by adaptively estimating the detection probability and clutter rate. The fact that MS-AGLMB-NC has a smaller OSPA than MS-GLMB-C indicates that the CPHD’s adaptive estimation of the detection probability and clutter rate is more effective in improving tracking accuracy than the group structure model.

As shown in Table 4, both the cluster structure model and the CPHD adaptive estimation parameters reduce OSPA, with the CPHD adaptive estimation yielding a more significant reduction; however, it also incurs a higher computational load. Compared to MS-GLMB-C, the CPHD adaptive parameter estimation in MS-AGLMB increases computation time by approximately 21%, but reduces OSPA by approximately 32%. This indicates that the computational complexity of our proposed MS-AGLMB is acceptable. MS-AGLMB-FP, due to its fixed high-clutter-to-signal-ratio parameter, also requires a longer computation time.

To investigate the impact of the number of Gibbs samples on accuracy and efficiency, we increased the number of Gibbs samples G in the full MS-AGLMB filter from 50 to 1000. For each setting, 100 Monte Carlo runs were performed under Scenario 3. As shown in Table 5, as the number of Gibbs samples increases, the OSPA error gradually decreases, and the number of mutually exclusive posterior hypotheses retained after pruning and merging increases; however, the computation time also increases accordingly. When the number of Gibbs samples exceeds 500, the improvement in tracking accuracy becomes minimal, while the computational cost increases significantly.

## 6. Conclusions

To address the problem of cluster target tracking under conditions of unknown detection probability and clutter rate, this paper proposes an MS-AGLMB filter. In real-world tracking scenarios, environmental factors can affect the detection probability and clutter rate of sensors. Traditional filters suffer from reduced tracking accuracy when prior parameters are unavailable. Cluster targets differ from general multi-target scenarios, and their cooperative interaction characteristics further degrade the filter’s tracking accuracy. In this paper, a virtual leader–follower model is employed to describe the dynamic interaction process among cluster members. An adaptive CPHD filter is used to estimate detection probability and clutter rate as parameters, and an improved GLMB filter is designed based on this approach. Furthermore, this paper employs the Gibbs sampling method to efficiently truncate the posterior GLMB hypotheses, thereby addressing the multi-sensor measurement partitioning problem, and achieves better tracking performance than other multi-sensor filters. Simulation results demonstrate that the MS-AGLMB exhibits excellent tracking performance for cluster targets in complex environments.

Beyond cluster target tracking, the core techniques developed in this paper—namely adaptive clutter estimation, multi-sensor fusion, and robust trajectory maintenance—have broader implications for modern multi-object tracking (MOT) systems. Recent deep learning strategies demonstrate that reliable multi-sensor tracking critically depends on robust uncertainty handling and sensor fusion design [9]. The adaptive CPHD-based parameter estimation scheme proposed here provides a principled Bayesian approach to online clutter and detection profile adaptation, which can complement data-driven association methods by reducing reliance on manually tuned thresholds and offline learned priors. Integrating the model-based RFS filtering framework with learned detectors and feature extractors represents a promising direction toward next-generation MOT systems that are both data-efficient and robust to changing environmental conditions. We hope the ideas presented in this paper will stimulate such cross-fertilization between classical Bayesian tracking and modern learning-based approaches.

## Figures and Tables

**Table 1:** Capabilities of Each Filter.

Filters	Filter Framework	Sensor $p_{i,D}$, and $\lambda_{c}$	Cluster Structuring	Sensor Fusion
Single-GLMB	GLMB	Known	Disabled	Disabled
DP-GLMB	GLMB	Unknown	Disabled	Enabled
MS-GLMB	GLMB	Known	Disabled	Enabled
MS-MeMBer	MeMBer	Known	Enabled	Enabled
MS-AGLMB	GLMB	Unknown	Enabled	Enabled

**Table 2:** Parameters for different scenarios.

Scenario	Detection Probability $p_{i,D}$	Average Clutter Rate $\lambda_{c}$
1	0.70	30
2	ranging from 0.70 to 0.90	ranging from 5 to 30

**Table 3:** Capabilities of Each Filter in Ablation Studies in scenario 3.

Filters	Cluster Structuring	Sensor $p_{i,D}$, and $\lambda_{c}$	CPHD
MS-GLMB-C	Enabled	Unknown true value	Disabled
MS-AGLMB-NC	Disabled	Unknown true value	Enabled
MS-AGLMB-TP	Enabled	Known true value	Disabled
MS-AGLMB-FP	Enabled	Fixed error value	Disabled
MS-AGLMB	Enabled	Unknown true value	Enabled

**Table 4:** Total error and operating time for each filter.

Filters	Total	Time
MS-GLMB-C	30.17	85.59
MS-AGLMB-NC	21.67	100.95
MS-AGLMB-TP	16.45	76.24
MS-AGLMB-FP	34.81	105.87
MS-AGLMB	20.38	104.27

**Table 5:** Filter performance at different Gibbs sampling rates.

Gibbs Samples	Total	Time	Retained Hypotheses
50	27.05	41.38	378
100	24.96	60.82	453
200	22.73	76.24	501
500	20.54	105.36	535
700	19.12	141.27	542
1000	18.55	190.63	548

## Data Availability

Data will be made available on request.

## Abbreviations

The following abbreviations are used in this manuscript:

GLMB	Generalized Labeled Multi-Bernoulli
LMB	Labeled Multi-Bernoulli
CPHD	Cardinalized Probability Hypothesis Density
PHD	Probability Hypothesis Density
MeMBer	Multi-Target Multi-Bernoulli
UKF	Unscented Kalman Filter
MRF	Markov Random Field
PMBM	Poisson Multi-Bernoulli Mixture
RHM	Random Hypersurface Model
OSPA	Optimal Sub-Pattern Assignment
FOV	Field of View
RFS	Random Finite Set
